# Natural Selection and Functional Potentials of Human Noncoding Elements Revealed by Analysis of Next Generation Sequencing Data

**DOI:** 10.1371/journal.pone.0129023

**Published:** 2015-06-08

**Authors:** Pankaj Jha, Dongsheng Lu, Shuhua Xu

**Affiliations:** 1 Chinese Academy of Sciences (CAS) Key Laboratory of Computational Biology,Max Planck Independent Research Group on Population Genomics, CAS-MPG Partner Institute for Computational Biology (PICB), Shanghai Institutes for Biological Sciences, Chinese Academy of Sciences, Shanghai, China; 2 School of Life Science and Technology, ShanghaiTec University, Shanghai, China; 3 Collaborative Innovation Center of Genetics and Development, Shanghai, China; Cincinnati Children's Hospital Medical Center, UNITED STATES

## Abstract

Noncoding DNA sequences (NCS) have attracted much attention recently due to their functional potentials. Here we attempted to reveal the functional roles of noncoding sequences from the point of view of natural selection that typically indicates the functional potentials of certain genomic elements. We analyzed nearly 37 million single nucleotide polymorphisms (SNPs) of Phase I data of the 1000 Genomes Project. We estimated a series of key parameters of population genetics and molecular evolution to characterize sequence variations of the noncoding genome within and between populations, and identified the natural selection footprints in NCS in worldwide human populations. Our results showed that purifying selection is prevalent and there is substantial constraint of variations in NCS, while positive selectionis more likely to be specific to some particular genomic regions and regional populations. Intriguingly, we observed larger fraction of non-conserved NCS variants with lower derived allele frequency in the genome, indicating possible functional gain of non-conserved NCS. Notably, NCS elements are enriched for potentially functional markers such as eQTLs, TF motif, and DNase I footprints in the genome. More interestingly, some NCS variants associated with diseases such as Alzheimer's disease, Type 1 diabetes, and immune-related bowel disorder (IBD) showed signatures of positive selection, although the majority of NCS variants, reported as risk alleles by genome-wide association studies, showed signatures of negative selection. Our analyses provided compelling evidence of natural selection forces on noncoding sequences in the human genome and advanced our understanding of their functional potentials that play important roles in disease etiology and human evolution.

## Introduction

It is important to understand the variations within DNA sequences because these are substrates of evolutionary and natural selection forces and the causal factors of many human diseases. In the human genome, a large proportion of DNA sequences consist of noncoding sequences (NCS) that do not code for functional proteins and are mostly often referred to as "Junk DNA". Despite the involvement of noncoding sequences in some important biological functions such as development, muscle differentiation, metabolic genes, oncogenes, evolution of tissue- and lineage-specific gene expression, and skeletal development, the main functions of them are unknown [1–4]. In this regard,the evolutionary and demographic forces leave traces in the genome that will enable us to investigate the functional potential of the genomic elements in the neutrally evolving genome and to determine the targets of natural selection [5, 6]. It is estimated that about 3–8% of the human genome is subject to evolutionary constraint, indicating that a large fraction of the genomeunderlying selection were contributed by NCS regions and potentially functional [7–9]. Furthermore, studies in primates and in other species such as *Drosophila* and rodents suggested natural selection as a driving force of the functionality of noncoding DNA sequences [10–12]. Earlier studies of the noncoding sequences in human populations have suggested that evolutionary and natural selection forces are important in shaping the biological functions of NCS elements in humans [13–15]. However, these studies were limited by small scale of sequencing data. The potential effects of noncoding sequences in the regulation of protein coding genes such as conserved noncoding DNA sequences, cis-regulatory regions, and miRNAs binding sites are suggestive of how natural selection played an important role in the evolution of these elements in humans [16–18].

Recently, the ENCODE project has provided the functional landscape of genomic elements and we estimated that nearly 80% of the human genome is biochemically functional in diverse human cell lines, especially those outside the known protein coding regions [19]. However, this estimation has also raised concern about the fraction of the human genome under evolutionary and natural selection forces [20]. Studies using a combined data of human sequencing data and ENCODE functional elements have suggested that NCS elements such as transcription factor binding sites and regulatory non-conserved regions underlie lineage-specific purifying selection and the gain of regulatory functions in recent human evolution [16, 21, 22]. Furthermore, the genomic variations within the noncoding sequences showed that the extent of selection is associated with the genomic property of each subclass of noncoding elements [23]. Indeed, population differentiation and selection of specific element such as miRNAs and its correlation with gene expression pattern in the human populations have been observed [24, 25]. Furthermore, the majority (more than 90%) of the genetic variants identified by genome-wideassociation studies (GWAS) were mainly associated with the noncoding part of the genome and might have transcriptional regulation effect or act as a quantitative trait loci affecting nearby expressed genes [26, 27]. Despite several numbers of studies on NCS sequences in the humans, a detailed investigation of noncoding sequences with respect to the evolutionary and natural selection forces has yet to be explored, both at the genome and at the population levels, especially in diverse human populations.

The purpose of this study is to understand the functional potential of noncoding DNA sequences by examining the genomic footprints left bynatural selection in the human genome. We hypothesized that the genetic differences in NCS elements in the neutrally evolving genome indicatetheir differential functional roles in the fitness of individuals in the population and targets of natural selection in the genome. Here, we analyzed sequence variants in diverse worldwide human populations using data obtained from the 1000 Genomes Project Phase I [28] and partitioned the genome broadly into (i) coding and noncoding parts of genes; 5′UTR, 3′UTR, intron, and CDS, (ii) noncoding RNAs (ncRNAs); miRNA, lincRNA, miscRNA, snoRNA, snRNA, tRNA and rRNA, (iii) pseudogene, and (iv) conserved noncoding sequences. We examined the relationship between a series of key population genetic and evolutionary parameters with genomic elements in worldwide populations. In the subsequent analyses, we looked for the enrichment of regulatory markers in NCS, such as expressed quantitative trait loci (eQTLs), TF motif, enhancers, and DNase I, and also examined natural selection signature in NCS variants reported by genome-wide association studies. Finally, we performed gene set enrichment analysis of eQTL associated positively with selected NCS variants in European populations to gain insights on the enrichment of expressed genes that are important for local adaptation.

## Materials and Methods

### Distribution and coordinates of genomic elements

In this analysis, we classified the genome into (i) genes in coding sequences (CDS) and noncoding sequences: 5′UTR, 3′UTR, and introns. The CDS element of a gene codes for functional proteins, while 5′UTR and 3′UTR are involved in transcriptional regulation functions such as promoter activity and down regulation of gene at the site of miRNA binding [29]. Intronic variants play important roles in the alternative splicing of a gene that causes multiple transcripts of a gene. (ii) intergenic regions, which are associated with many disease variants; (iii) pseudogenes, which are redundant copies of genes that lost their potential for functional products [30]; (iv) noncoding RNAs (ncRNAs), including microRNA (miRNA), tRNA, ribosomal RNA (rRNA), small nucleolarRNA (snoRNA), miscellaneous RNA (miscRNA), small nuclear RNA (snRNA), and long intergenic noncoding RNA (lincRNA). Most of these ncRNAs have functional roles such as miRNA in gene regulation after its binding to 3′UTR region of a gene [25], lincRNA in gene regulation [2], tRNA and rRNA in translation process [31], snRNA and snoRNA in spliceosomal removal of pre-mRNA introns and nucleolar maturation of cytoplasmic ribosomal RNAs, respectively [32], and (v) conserved noncoding sequences (CNC). To obtain the genomic coordinates, we used several databases according to the type of noncoding sequences. The validated miRNA coordinates were retrieved from miRbase database (http://www.mirbase.org/; miRBase version 18). Coordinates for rRNA, snoRNA, snRNA, and miscRNA were retrieved from Ensembl annotation 66 (www.ensembl.org). Coordinates for pseudogenes were obtained from pseudogene database (http://www.pseudogene.org). Coordinates for 5′UTR, 3′UTR, introns, and CDS were obtained from Ensembl annotation 66. For 3′UTR, 5′UTRs, introns, and CDS, we used the longest transcript of a gene. Coordinates for lincRNA (only stringent lincRNAs were used here) were obtained from Human Body Map for lincRNA (http://www.broadinstitute.org/genome_bio/human_lincrna/) [33]. Genome-wide markers and intergenic regions were also used in this analysis. For intergenic region, 10 kb sequences apart from any studied genomic elements were used and intergenic regions larger than 10 kb without any variant were removed from this analysis. We also used conserved noncoding sequences (CNC) regions, conserved across the mammalian species identified by Pritchard’s lab [34]. Genomic coordinates from all the sources were converted into hg19 built using LiftOver in this study. Gene annotation was done according to UCSC hg19 (GRCh37/hg19) built (http://genome.ucsc.edu/). In total, nearly 37 million single nucleotide polymorphisms (SNPs) of the 1000 Genomes Project Phase I data were analyzed.

### Genotype data from the 1000 Genomes Project Phase I

All the SNP genotype data were retrieved from the 1000 Genomes Project Phase I data release (http://www.1000genomes.org/) [35]. A total of 1,092 samples were obtained for all the elements, including 61 ASW (African ancestry in Southwest USA), 85 CEU (Utah residents with Northern and Western European ancestry), 97 CHB (Han Chinese in Beijing, China), 100 CHS (Han Chinese from South China), 60 CLM (Colombian in Medellin), 93 FIN (Finnish), 89 GBR (British), 14 IBS (Iberian from Spain), 89 JPT (Japanese in Tokyo, Japan), 97 LWK (Luhya in Webuye, Kenya), 66 MEX (Mexican ancestry in Los Angeles, California), 55 PUR (Puerto Rican), 98 TSI (Toscans in Italy), and 88 YRI (Yoruba in Ibadan, Nigeria). For this analysis, we used a total of 36,820,992 SNPs restricted to autosomal chromosomes (chromosome-wise distribution is given in S1 Table). The length distribution and SNP density of each element are shown in S1 Fig and S2 Fig, respectively. For some analyses, we pooled some of the populations based on the three ancestries, i.e., for Europeans, we pooled CEU, FIN, GBR, and TSI; for Africans, we pooled YRI and LWK; and for Asians, we pooled CHB and JPT.

### Estimation of population genetic parameters

We used the phased data from the 1000 Genomes Project to calculate the summary statistics for testing the neutrality of each element. Sequence-based neutrality test such as number of segregation site (*S*), Tajima’s *D* [36], Fu and Li’s F* [37], and Fay and Wu’s *H* [38] were analyzed using Variscan software version 2.0.3 [39]. The nucleotide diversity (π) was also calculated within populations as the average of the pairwise nucleotide differences per site between any two sequences. First, the summary statistics were calculated using concatenated sequences of each type of element and then calculated for each element in 10 kb of window sizes with a 5 kb sliding window. As the sample size of the concatenated windows was insufficient for statistical inference,the mean Tajima’s *D* value of these concatenated 10kb windows were then recalculated using bootstrapping approach that repeated for 1000 times, and based on these, the mean value was calculated. The permutation test between genome-wide and any other element was performed, and after repeating this process 10,000 times, theproportion difference of the means larger than the original observed difference wasdesignated as the *P*-value of this permutation test. We used this approach to calculate the neutrality tests for genome-wide dataset and used this result as a background control because in a neutrally evolving genome, this estimate can give the overall demographic effect in a population. In this study, all the ancestral allele information was obtained from the 1000 Genomes Project (http://www.1000genomes.org/). Population differentiation (*F*_ST_) of each SNP for all the elements was calculated using in-house Perl script based on Weir and Cockerham’s formula [40]. Based on the three continental populations (CEU, CHB, and YRI) taken together, the global *F*_ST_ for each variant was calculated. Furthermore, for pair-wise *F*_ST_ analysis, we pooled the populations based on their ancestries, namely Asians (ASN), Europeans (EUR), and Africans (AFR) (as revealed by principal component analysis and STRUCTURE analysis) and calculated the pairwise *F*_ST_ between these grouped populations. We also calculated minor allele frequency (MAF) and used the ancestral allele information to calculate the derived allele frequency (DAF).

### Population structure analysis

For population structure analysis, we used STRUCTURE, a Bayesian clustering method implemented for inferring population structure for all elements separately among all the individuals [41]. For principal component analysis (PCA), we used the EIGENSOFT v.2 package [42], with default parameters, to calculate up to 10 eigenvectors. As the number of variants varied among the studied elements, we resampled the smallest set of 870 SNPs based on the markers in rRNA sequences which have the smallest number of markers in dataset, and ran STRUCTURE and PCA to overrule the bias in estimates that may arise due to the differences of SNPs in each element studied.

### Functional constraint analysis based on evolutionary conservation

Sequence conservation across species is one of the consequences of purifying selection due to functional constraint. To estimate the conservation in the sequences, we obtained GERP (Genomic Evolutionary Rate Profiling) scores [9] from the UCSC genome browser (hg19) and mapped them with each element sequences. We adopted a GERP threshold of ≥ 2 and ≥ 3, and estimated the percentage of sequence length in each element for conservation. We classified SNPs into conserved (GERP ≥ 2 and ≥ 3) and non-conserved (GERP ≤ 2 and ≤ 3) and examined for lower DAF ≤ 0.05 in both categories of each elements.

### Evaluation of statistical significance

The statistical significant (*P* value) was obtained by Pearson’s chi-square test with 1 degree of freedom (significant threshold was *P* < 0.05). Fisher exact test was also employed for datasets with small sample size. Enrichment analysis was conducted by calculating the ratio between observed values (P_O_) divided by expected values (P_E_) in our dataset.

### Detection of positive selection signalsin NCS

Due to the genetic hitchhiking, the frequency of flanking variants around the core variant also increases. Thus iHS statistics were calculated for a given core SNP in a population for detecting such events [43]. The unstandardized iHS (integrated haplotype score) is defined as ln(iHH_A_/ iHH_D_) where iHH_A_ and iHH_D_ are defined as integrated EHH (extended haplotype homozygosity) scores for ancestral and derived allele, respectively. We used the default parameter to calculate the iHS score [43] with rehh package implemented in R [44].

Finally, to identify the signature of positive selection underneath the noncoding sequences, we used all of the three parameters, namely derived allele frequency, population differentiation (*F*_ST_), and iHS score. A threshold was set for each statistics; i) DAF > 0.5, ii) *F*_ST_ > 0.3, and ii) |iHS| > 2, and variants that fulfilled any two categories were listed and annotated near to 5 kb flanking or overlapping genes.

### RegulomeDB and NCS variants

We examined the enrichment of NCS variants for their functionalities using the recently created database for regulatory elements in the human genome in RegulomeDB (http://regulome.stanford.edu/) [45]. We mapped our entire dataset with RegulomeDB and annotated all the variants that fell within the regulatory regions and further classified them into six classes (1 to 6, we pooled the subclasses in each class for the ease of analysis) according to the database classification and searched for the significant enrichment of NCS variants compared with CDS variants.

### GWAS-associated variants and NCS elements

We also investigated the enrichment of NCS variants with genome-wide association database (GWAS) in our study and obtained the information for all the studied SNPs that are present in GWAS catalogue (www.genome.gov/gwastudies). We further categorized the mapped datasets into three parts: i) SNPs that were replicated internally, ii) SNPs that were replicated externally, and iii) SNPs not replicated (NR) as classified by Maurano *et al*. [26].

### eQTL identification and pathway enrichment analysis

NCS variants that are positive for selection based on DAF, *F*_ST_, and iHS values were further used for the identification of associated expression quantitative traits (eQTLs) genes. We used eQTL Browser (http://eqtl.uchicago.edu/cgi-bin/gbrowse/eqtl/) and extracted genes associated with selected variants in CEU populations. Next, a multigene feature based enrichment analysis was performed using ToppCluster (http://toppcluster.cchmc.org/) [46], which uses all the available resources related to the given genes. Statistically significant enrichment (false discovery rate; FDR correction at a cut-off *P* value < 0.05) information was retrieved, and enriched pathways were further plotted with Cytoscape v2.8.3 software [47] for visualization.

Apart from the above analyses, we also used HaploReg v2 for obtaining the Linkage Disequilibrium (LD) (http://www.broadinstitute.org/mammals/haploreg/haploreg.php) which has information of significant variants and encompassing regions, and other information such as position wait matrix (PWM) log odds score of regulatory motifs for reference and alternative allele sequences. Also, regulatory markers peak scores of H3K4me1, H3K4Me3, H3K27Ac, and DHS for different tissues were obtained from UCSC table browser of selected variants regions (http://genome.ucsc.edu/).

## Results

### Natural selection implied by neutrality analysis of noncoding sequences

The summary statistics based on site frequency spectra are potentially informative in testing the goodness-of-fit of the standard model of neutrality,in that any significant deviation from the neutral model reflects either natural selection or some special population demographic events occurred in the population studied [6, 36–38]. Complete DNA sequencing data in multiple individuals from diverse worldwide human populations provided robust estimation of demography as well as natural selection experienced by modern humans.

In our dataset, the sequence length of genomic elements varied greatly from the smallest for tRNA (0.038 mega-base (Mb)) to the largest for intron (1003.7 Mb), while the SNP density was found to be the lowest for CDS (0.0044) and the highest for tRNA (0.0119) in the genome (S1 Fig; S2 Fig; and S1 Table). These differences could affect the estimation of sequence based neutrality tests and subsequent interpretations. To overcome these effects on neutrality tests, we calculated the statistics and performed the test by sampling data with bootstrapping from 10 kb regionsto 5 kb sliding windows.

**Fig 1:**
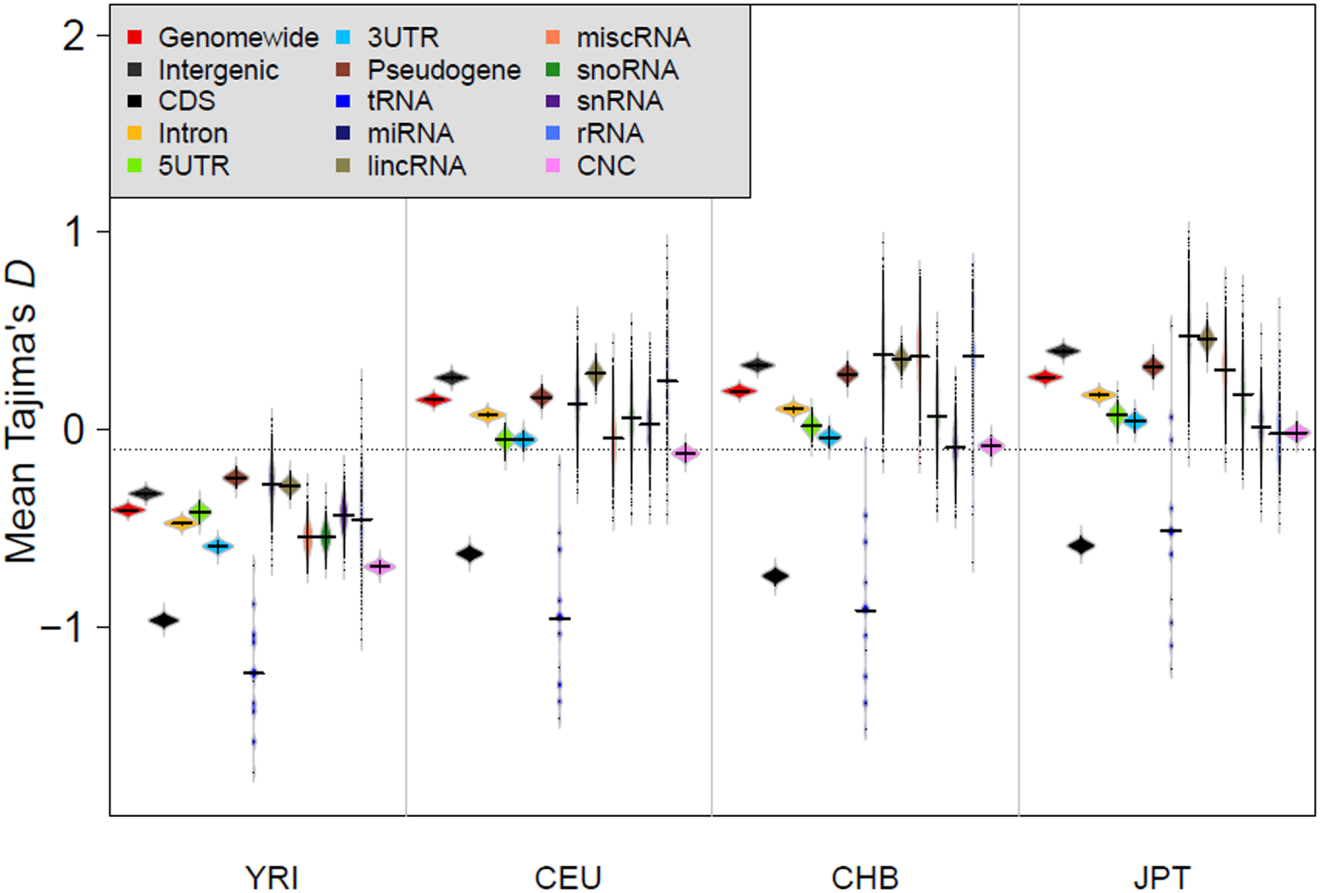
Beanplot of Tajima’s *D* estimate distribution of genomic elements in YRI, CEU, CHB, and JPT populations. Comparison between populations revealed a lower *D* distribution in YRI populations with respect to CEU, CHB, and JPT. Within population comparison showed the lowest mean *D* for tRNA in the genome (even less than that for CDS) indicating that tRNA is strongly under purifying selection in the genome. However, mean *D* was observed to be higher for pseudogene, intergenic, lincRNA, and miRNA in the genome. The overall mean *D* showed lower value for most of the genomic elements in the genome.

First, we estimatedthe genome-wide Tajima’s *D* and observed that the distribution was heavily skewed towards positive value for all the populations studied, and towards the negative value in non-African populations (S3 Fig). These results reflected evidence of severe bottleneck experienced by worldwide populations in the past and recent population expansion of non-African populations since “Out of Africa” [48]. In addition, the mean Tajima’s *D* values of all the studied elements were lower in the African populations (YRI) than in non-African populations, showing that NCS elements have experienced similar demographic forces tothe entire genome (Fig 1). However, the element-wise comparison within population showed that the mean Tajima’s *D* value of tRNAs was the lowest among the studied elements and significantly lower compared to the genome-wide control (permutation test; *P* < 0.05). The mean Tajima’s *D* values for CDS, intron, 3′UTR and CNC were significantly lower than the genome-wide background in YRI, CEU, and CHB but not in JPT (permutation test; *P* < 0.05) (S2 Table). Also, the mean Tajima’s *D* was significantly lower for 5′UTR except for YRI populations, while intergenic and lincRNA showed significantly higher values for Tajima’s *D* in all the populations compared to the genome-wide values. Pseudogenes also showed higher mean Tajima’s *D* value except for the CEU populations. The other ncRNA elements, snoRNA, snRNA, rRNA, and miscRNA showed lower Tajima’s *D* values compared to the genome-wide ones but this difference was not statistically significant. Analysis using other statistic measures for neutrality such as Fu and Li’s D* and Fu and Li’s F* showed a similar pattern (S2 Table).

We also estimated the mean nucleotide diversity (π) which showed the highest value for tRNAs but the lowest for CDS (0.0009 (±0.0002) for tRNA and (0.0004±0.0003) for CDS in CHB) (S4 Fig and S2 Table). However, the diversity value for tRNAs was not much different from that of the genome-wide in all the four populations based on permutation test(S2 Table). The high value of diversity and low value of Tajima’s *D* for tRNAs suggest that tRNAs are under functional constraint but tend to be weak, thus many variants are in low frequency without being entirely eliminated from the population. These differences can also be explained by tRNA gene diversity, where the number of tRNA genes is higher than the number of isoacceptors [49]. The diversity of CDS, 5′UTR, 3′UTR, intron, and CNC was significantly lower than that for the genome-wide, whereas the diversity of lincRNA and intergenic elements was higher than that for the genome-wide (permutation test; *P* < 0.05; S2 Table). Interestingly, the diversity of pseudogenes was significantly lower than that of the genome-wide in all the four populations (permutation test; *P* < 0.05). In addition, the deviation from the neutrality of a particular category of elements could have resulted from population structure. However, we did not see differences in inferences of ancestry of individuals for different genomic elements based on STRUCTURE and PCA (S5 Fig and S6 Fig). The observation was also consistent with previous reports [28]. However, we observed noise in ncRNAs in STRUCTURE plots and addressed this issue by calculating and comparing observed heterozygosity (*H*o) of genomic elements. We observed that *H*o was higher with ncRNAs than with other elements (S7 Fig). We grouped the populations according to the genetic clustering patterns revealed by STRUCUTRE and PCA for further analysis.

### Footprints of purifying selection revealed by allele frequency spectrum of NCS elements

**Fig 2:**
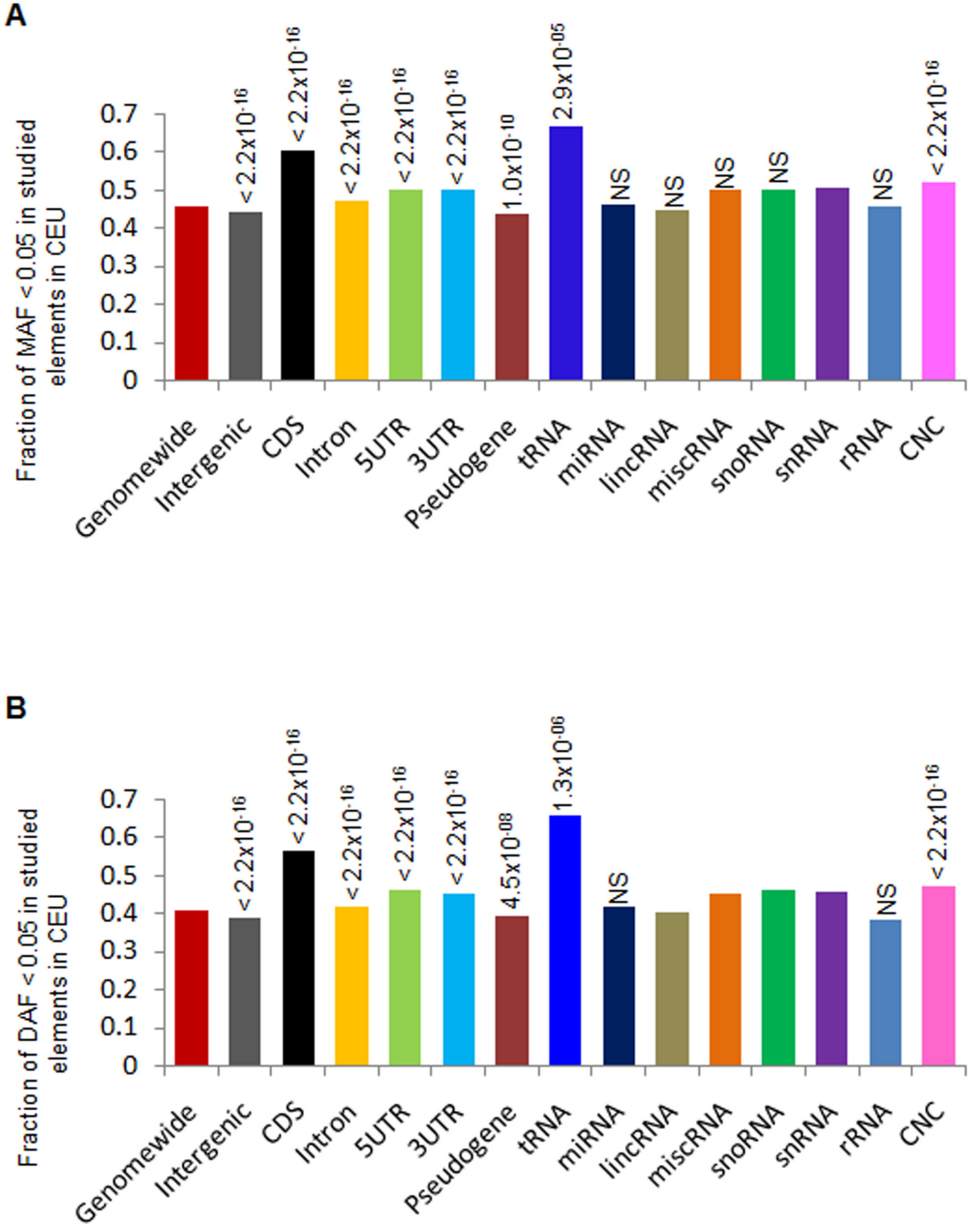
Enrichment of minor allele frequency (MAF) < 0.05 (A) and derived allele frequency (DAF) < 0.05 (B) of studied elements in CEU populations. In the genome, intron, CDS, 5′UTR, 3′UTR, tRNA, and CNC had significantly enriched rare variants compared to the genome-wide background, whereas pseudogene and intergenic elements showed significant depletion of these variants. ncRNAs showed a high number of low MAF variants over the genome-wide background but this difference is not significant(Significance; χ^2^ test, *P* < 0.05, NS, stands for non-significant).

We further analyzed minor allele frequency (MAF) and derived allele frequency (DAF) spectrum to search for signatures of natural selection in the population. In the genome, a large fraction (30–60%) of variants having MAF value was within 0.05 in worldwide human populations across the studied elements (S8 Fig). These observed highfraction of variants having low MAF value suggested the possibility of natural selection or some demographical events such as population expansion [50]. In our analysis, tRNAs showed the highest MAF fraction (60%) falling within a frequency of less than 5%, a value that is higher than that for CDS. This observation is consistent with that from a previous study [23] and is in accordance with our highly skewed lower Tajima’s *D* value for tRNAs in the genome. We also quantified the low MAF variants < 0.05 for each element in the four major populations (CEU, CHB, JPT, and YRI) and compared the result with that obtained from the genome-wide background (Fig 2A and S11A–S11C Figs). We observed that a high number of low MAF variants were significantly enriched for many elements. For example, the CEU populations exhibited an excess of low MAF variants in tRNA, CDS, 5′UTR, 3′UTR, intron, and CNC but a reduction in pseudogene and intergenic elements, when compared to the genome-wide background (χ^2^ test, *P* < 0.05) (Fig 2A). The element-wise comparison showed a consistently higher fraction of variants in the lower MAF bin for tRNA, CDS, 5′UTR, 3′UTR, intron and a significant depletion in pseudogene and intergenic elements in all the four populations analyzed. However, population specific enrichment of some of the ncRNAs was observed in these populations (S9A–S9C Figs). Occurrence of lower DAF (DAF < 0.05) was similar in pattern to that of MAF spectrum in worldwide human populations (S10 Fig). Similarly, element-wise comparison showed significant enrichment of lower DAF in tRNA, CDS, 5′UTR, 3′UTR, intron, and CNC and depletion of pseudogene and intergenic elements compared to the genome-wide background (Fig 2B and S11A–S11C Figs). Again, higher proportion of lower DAF was also observed in ncRNAs, such as in CEU; snRNA, snoRNA, and miscRNA showed higher fraction of low DAF compared to the genome-wide and the depletion of lincRNA, but were not significant (Fig 2B). The high proportion of lower MAF and DAF variants in NCS elements being similar to coding sequences but different from the genomewide pattern indicates that functional constraint and purifying selection are acting on these elements.

### Deviation from Neutrality of NCS elements by between Population Comparison

Natural selection usually leads to much differential allele frequency between populations. We further used the *F*_ST_ statistics to substantiate our natural selection forces acting on NCS elements. First, we estimated the global *F*_ST_ of elements and observed that the genome-wide *F*_ST_ was 0.1367 among Asian, European and African populations. Furthermore, within element comparison revealed the highest differences for miscRNA (0.1557) and the lowest for tRNA (0.1050), while CDS was 0.1358 among the continental populations (S12 Fig). In addition, the pair-wise *F*_ST_ differences between ASN-EUR werelower compared to AFR-ASN populations at the genome-wide scale as well as for studied elements, indicating higher genetic differences between African and Asian populations or recent gene flow between African and European populations (S13 Fig).

**Fig 3:**
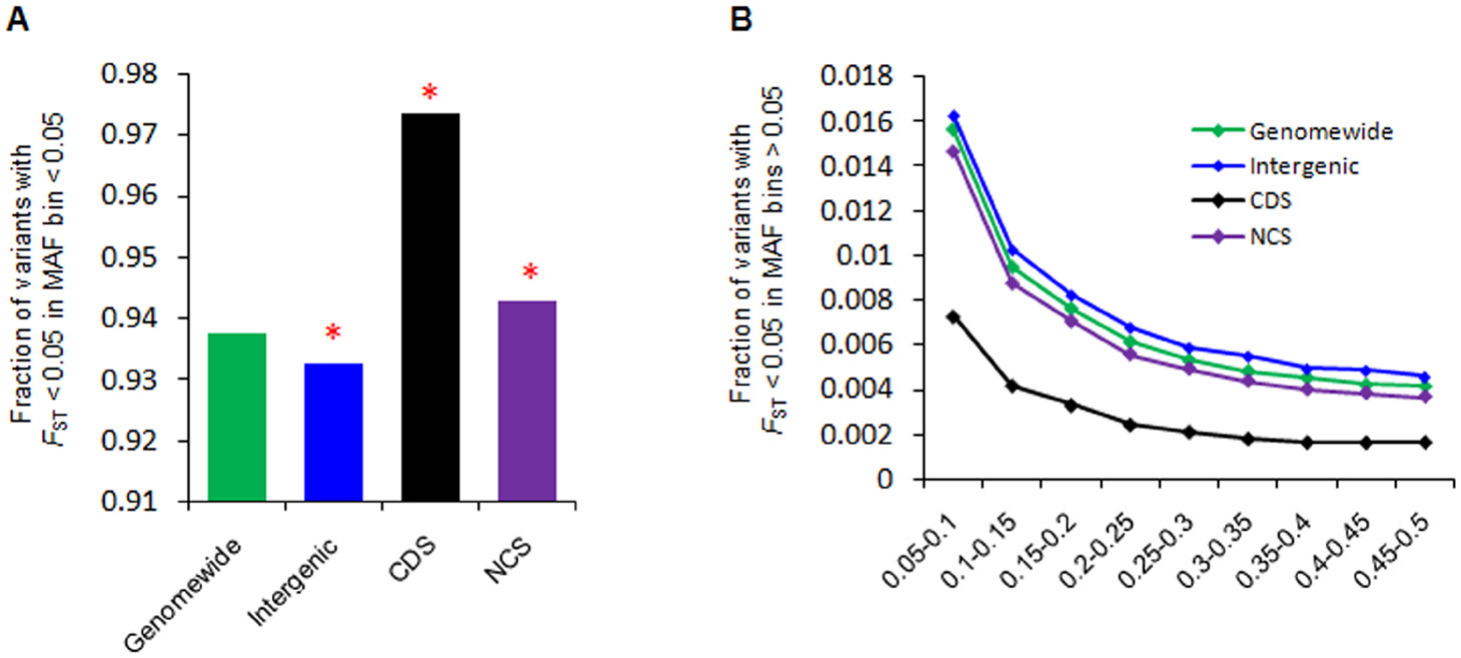
Signature of purifying selection in NCS elements. (A) A high number of low *F*_ST_ variants (< 0.05) were significantly enriched for CDS and NCS elements compared to the genome-wide background (red astral indicates significance; χ^2^ test *P*< 0.05). However, intergenic elements showed significant depletion for low *F*_ST_ variants. (B) Furthermore, low *F*_ST_ variants (< 0.05) of CDS and NCS in all high MAF bins were significantly less than those in the genome-wide background, indicating that NCS was under purifying selection just like CDS, although intergenic elements showed a significant high number of low *F*_ST_ variants in higher MAF bins.

We then analyzed the low *F*_ST_ variants in NCS elements because in the case of purifying selection, such variants will show enrichment in an element [51]. Our element-wise comparison of variants with *F*_ST_ value < 0.05 revealed a significant high number of such variants for intron, 3′UTR, tRNA, CDS and CNC and depletion for intergenic and pseudogene, when compared to the genome-wide background (χ^2^ test, *P* < 0.05) (S14 Fig). Again, such low *F*_ST_ variants in miscRNA, snoRNA, snRNA, and rRNA were not significant. For better comparison, we pooled all the NCS elements and classified low *F*_ST_ variants into four groups (genome-wide, intergenic, CDS and NCS) and compared these groups in different MAF bins. First, low *F*_ST_ variants with low MAF (0.05) showed significant enrichment for both CDS (χ^2^ test, *P* < 2.2x10^-16^) and NCS (χ^2^ test, *P* < 2.2×10^–16^) element but depletion for intergenic elements (χ^2^ test, *P* < 2.2×10^–16^), when compared with the genome-wide background (Fig 3A). In the intermediate minor allele frequency bins (Fig 3B), NCS and CDS showedsignificant depletion of low *F*_ST_ variants compared to the genome-wide variants (χ^2^ test, *P* < 0.05). While, intergenic elements showed a significant high number of low *F*_ST_ variants in intermediate MAF bins compared to the genome-wide control (χ^2^ test, *P* < 0.05). The excess of low *F*_ST_ variants in low MAF bin and depletion of low *F*_ST_ variants in intermediate allele bins further suggested the action of purifying selection that might have occurred in NCS regions.

### Non-conserved NCS Sequences and their Potential Functional gain

We extended our analysis into mapping studied elements for GERP conservation score of each nucleotide obtained from UCSC browser. We set the cutoff of GERP score to ≥ 3 and ≥ 2 and estimatedthe fraction of sequence length under conservation for determining purifying selection or evolutionary constraint. We observed 57–66% of CDS with such scores (GERP ≥ 3 and ≥ 2, respectively) compared to 51–67% of CNC sequence (Table 1). At the higher GERP score ≥ 3, CDS were significantly enriched compared to CNC sequences (χ^2^ test, *P* < 2.2×10^–16^). In addition, nearly 30% of tRNA, 5′UTR, 3′UTR, and miRNA sequence bases had scores greater than 2. Interestingly, with the higher GERP score of 3, miRNA showed significant enrichment for conservation, with ~22% of sequences compared to 20% of 5′UTR and 3′UTR sequences (χ^2^ test, *P* < 2.2×10^–16^). These results suggested that miRNA has more functional constraints compared to UTRs in the genome at a higher conservation level. Furthermore, we observed a very small fraction (0.7–5%) of sequences conservation for snRNA, rRNA, miscRNA, and pseudogenes. With a cutoff of GERP ≥ 3 and 2in our dataset, 4.3 and 9.7% of the genome, respectively, are functionally constrained, of which the majority are NCS [7, 9]. We also analyzed the lower DAF (derived allele frequency, DAF ≤ 0.05) variants in conserved (GERP ≥ 2 and 3) and non-conserved (GERP ≤ 2 and 3) regions of each type of elements. In the conserved region, we observed that 83–93% of CDS and CNC variants show conservation with lower DAF, while only 25% of rRNA fell within this category (S15A Fig). In ncRNAs, tRNA has the highest occurrence of variants with 93% showing conservation with lower DAF. In non-conserved region dataset, 85% of CDS and 80% of CNC variants exhibited lower DAF compared to 76% of non-conserved variants with lower DAF in the remaining NCS elements (S15B Fig). This observation of fraction of lower DAF variants in non-conserved regions indicates purifying selection though lower than conserved regions. This could be due tothe functional gain of these sites in the recent history of human evolution [22].

**Table 1:** Sequence conservation estimation based on GERP score in genomic elements.

Element	Total length	Length with score GERP ≥ 2	% of Length GERP ≥ 2	Length with score GERP ≥ 3	% of Length GERP ≥ 3
**Intergenic**	677004463	44825932	6.62	15447978	2.28
**CDS**	31236181	20831516	66.69	18025129	57.71
**Intron**	1003794319	116327120	11.59	47019014	4.68
**5UTR**	5454261	1701964	31.20	1040037	19.07
**3UTR**	25528453	7886772	30.89	5143298	20.15
**Pseudogene**	23725392	1246416	5.25	499373	2.10
**tRNA**	38174	13256	34.73	5818	15.24
**miRNA**	114425	34028	29.74	25953	22.68
**lincRNA**	4995724	548954	10.99	239455	4.79
**miscRNA**	175287	6041	3.45	2925	1.67
**snoRNA**	160173	24369	15.21	18049	11.27
**snRNA**	198739	3886	1.96	1422	0.72
**rRNA**	56531	1093	1.93	451	0.80
**CNC**	20248386	13646400	67.40	10378096	51.25
**Genomewide**	3095677412	300527849	9.70	132292161	4.27

### NCS elements and regional positive selection

**Fig 4:**
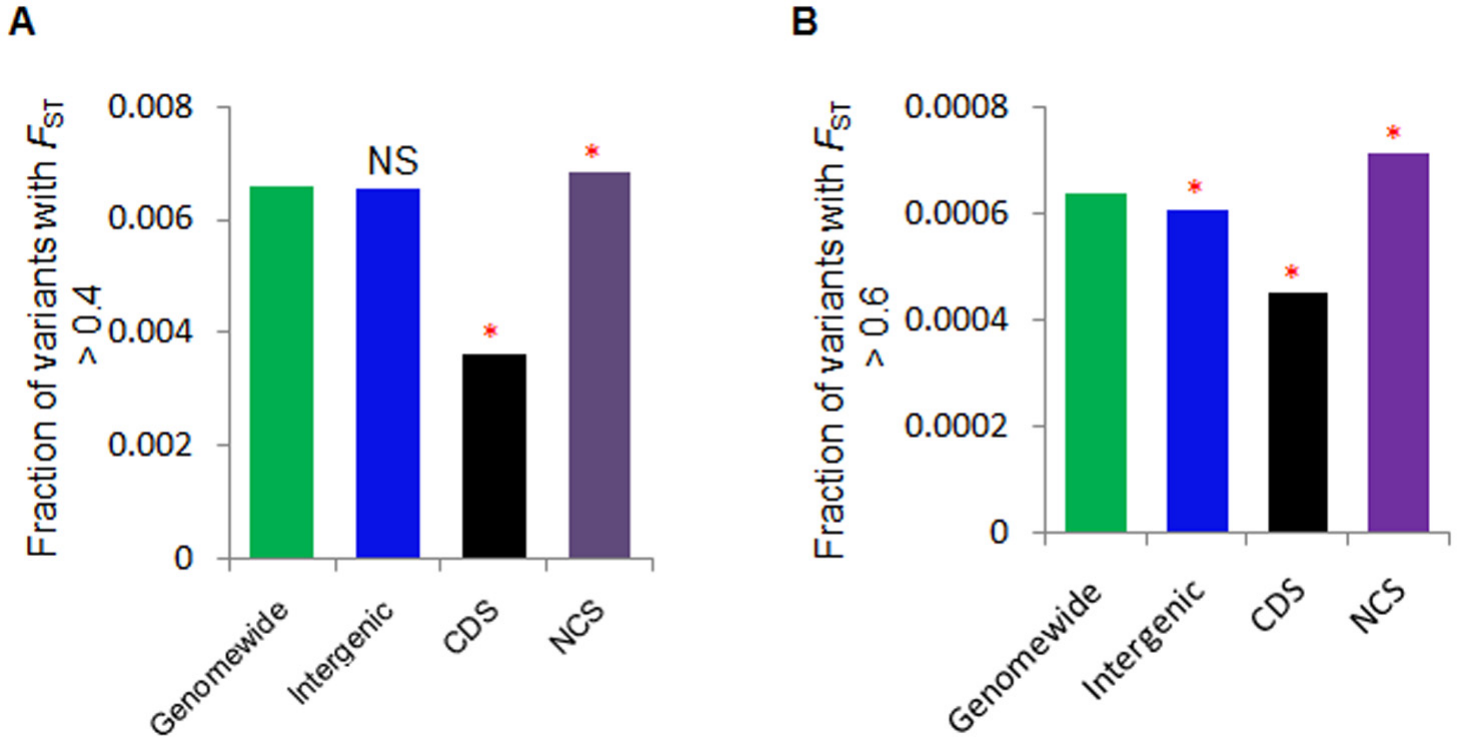
Enrichment of high *F*_ST_ NCS variants in the human genome for local positive selection. We detected a significant high number of high *F*_ST_ variants in NCS but a depletion of these variants in CDS, when compared to the genome-wide control at different *F*_ST_ levels (A) at > 0.4 (B) at > 0.6. A significant higher number of high *F*_ST_ variants were enriched in NCS regions than in CDS, only 12 in number at 0.8 (C).

We sought to understand the potential effect of local positive selection on sets of NCS variants in the human genome. To achieve this, we examined the enrichment of high *F*_ST_ variants > 0.4 in all the studied elements. Interestingly, we observed that with respect to the genome-wide background, intron (χ^2^ test, *P* < 2.2×10^–16^) and pseudogene (χ^2^ test, *P* = 0.002) showed a significant high number of high *F*_ST_ variants and depletion for CDS (χ^2^ test, *P* < 2.2×10^–16^) and CNC (χ^2^ test, *P* = 4.1×10^–16^) regions (S16 Fig). However, high *F*_ST_ variants in 3′UTR, lincRNA, miscRNA, snoRNA, and snRNA were not statistically significant compared to the genome-wide background. Tofurther analyze the genomic regions with a significant high number of high *F*_ST_ variants (> 0.4 and > 0.6), we pooled all NCS elements excluding intergenic element and compared NCS, intergenic, and CDS elements to the genome-wide background. Based on this analysis, we observed that NCS elements were significantly enriched compared to the genome-wide background (χ^2^ test, *P* < 2.2×10^–16^) in both of the high *F*_ST_ groups, while CDS were significantly depleted (χ^2^ test, *P* < 2.2×10^–16^) in *F*_ST_ larger than 0.4 and 0.6 (Figs 4A and 4B). Also, intergenic regions showed non-significant depletion in *F*_ST_ > 0.4 and significant depletion in *F*_ST_ > 0.6 (χ^2^ test, *P* < 2.2×10^–16^) compared to the genome-wide background. We further examined high *F*_ST_ variants > 0.4 in all MAF bins. The NCS elements showed significant enrichment for high *F*_ST_ variants in high MAF bins (> 0.3) (χ^2^ test, *P* < 0.05) compared to the genome-wide, while CDS showed enrichment only in MAF bins between 0.25 to 0.35 (χ^2^ test, *P* < 0.05) and depletion in higher MAF bins (> 0.35) (S17A Fig). However, intergenic elements showed depletion of high *F*_ST_ variants in high MAF bins (> 0.3) (χ^2^ test, *P* < 0.05). In higher *F*_ST_ variants > 0.6, NCS elements showed significant enrichment in high MAF bins (0.3–0.35 and from 0.4 to 0.5) while CDS and intergenic elements were not enriched in any MAF bins (S17B Fig).

We also looked for signatures of positive selection using integrated haplotype score (iHS) statistics by Voight *et al*. [43]. African populations showed the highest fraction of variants with |iHS| > 2 while Asian populations showed the lowest; an observation consistent with previous study [43] (S18A Fig). Our comparison of genomic elements revealed that the variants of |iHS| score > 2 were enriched in NCS regions (χ^2^ test, *P* < 2.2×10^–16^), and depletedin CDS (χ^2^ test, *P* < 2.2×10^–16^) compared to the intergenic variants (S18A Fig). Interestingly, we observed a considerable number of small ncRNAs variants with |iHS| ≥ 2 in worldwide human populations (S19 Fig). We further examined the enrichment of variants with |iHS| ≥ 2 and high *F*_ST_ > 0.3 in the genome and observed that NCS elements have again a larger number of such variants compared to CDS (S18B Fig). Thisshowedthat NCS variants play important roles in the local adaptations of modern humans compared to CDS variants. Finally, based on *F*_ST_ values > 0.3 and |iHS| ≥ 2, we retrieved all the SNPs from all the elements, and analysis of that dataset revealed the enrichment for CDS, intron, 5′UTR, 3′UTR, pseudogene, lincRNA, and CNC elements (top 1% of the variants). For miRNA, snRNA, snoRNA, rRNA, tRNA, and miscRNA, we analysed the variants with *F*_ST_ > 0.3 and |iHS| ≥ 2 and combined all the datasets for regulatory element and gene annotation (overlapping or nearby genes).

### Enrichment of regulatory elements in NCS

**Fig 5:**
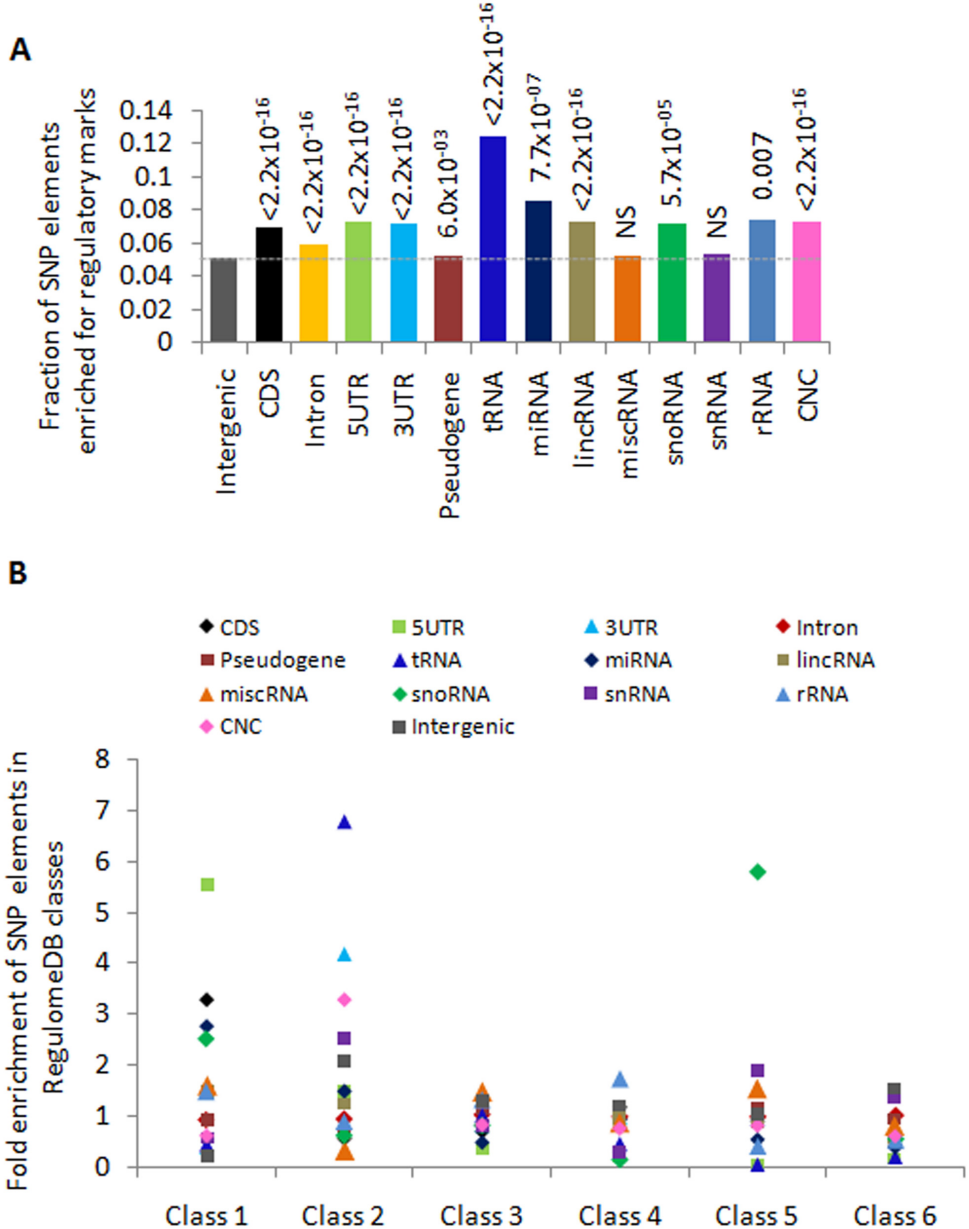
Enrichment of regulatory elements in the human genome. **(A)** In the genome, the number of variants that fell in various types of regulatory elements was significantly higher in NCS elements than in CDs. (B) Enrichment of the various genomic elements in different classes of RegulomeDB in the human genome. Class 1is the most enriched for regulatory elements including eQTLs and exhibited enrichment for 5′UTR. To calculate the enrichment, the observed value (fraction of SNPs observed in each class) was divided by the expected value (fraction of SNPs in each element presented in our dataset) at the genome-wide level.

Variations within protein coding genes, especially mutations that lead to changes in the amino acids, have been widely studied. However, the majority of variants associated with diseases or phenotypesare present in noncoding regions. Thus we extended our analysis for functional enrichment of coding and noncoding variants, and obtained all regulatory information using RegulomeDB [45]. Our data showed a significant high number of regulatory variants in all elements except for miscRNA and snRNA compared to intergenic variants (Fig 5A). Also, NCS elements, 3′UTR (χ^2^ test, *P* = 0.005), 5′UTR (χ^2^ test, *P* = 3.7×10^–7^), tRNA (χ^2^ test, *P* = 4.9×10^–14^), miRNA (χ^2^ test, *P* = 0.042), lincRNA (χ^2^ test, *P* = 0.001), and CNC (χ^2^ test, *P* = 3.7×10^–7^) had a significant high number of regulatory variants compared to CDS region. However, intron, pseudogene, miscRNA, snRNA, and intergenic elements showed significant depletion compared to CDS (χ^2^ test, *P* < 0.05), indicating a minimal regulatory potential of these elements. We also classified all mapped variants into six broad categories described by RegulomeDB and further looked for the enrichment of different SNP elements in the human genome. We observed that 5′UTR was more than 2-fold enriched compared to CDS in class 1, while 3′UTR and tRNA were enriched by 3-fold and 6-fold, respectively, when compared to CDS in class 2 (Fig 5B). We also observed the enrichment of snRNA (2-fold) and CNC (3-fold) variants in class 2, although the enrichment for 5′UTR was decreased in class 2 and in subsequent classes. Because the class 1 category encompasses major regulatory variants associated with the expression of target genes (eQTLs) and transcription factor binding motifs (TFBS), the enrichment of 5′UTR in this class indicated important functional region in the genome, consistent with the function of 5′UTRin gene regulation. These observations suggest that these enriched regulatory variants within NCS sequences may play important roles in adaptation and human diseases compared to coding regions in diverse human populations [52].

### GWAS associated NCS variants underlying purifying selection

**Fig 6:**
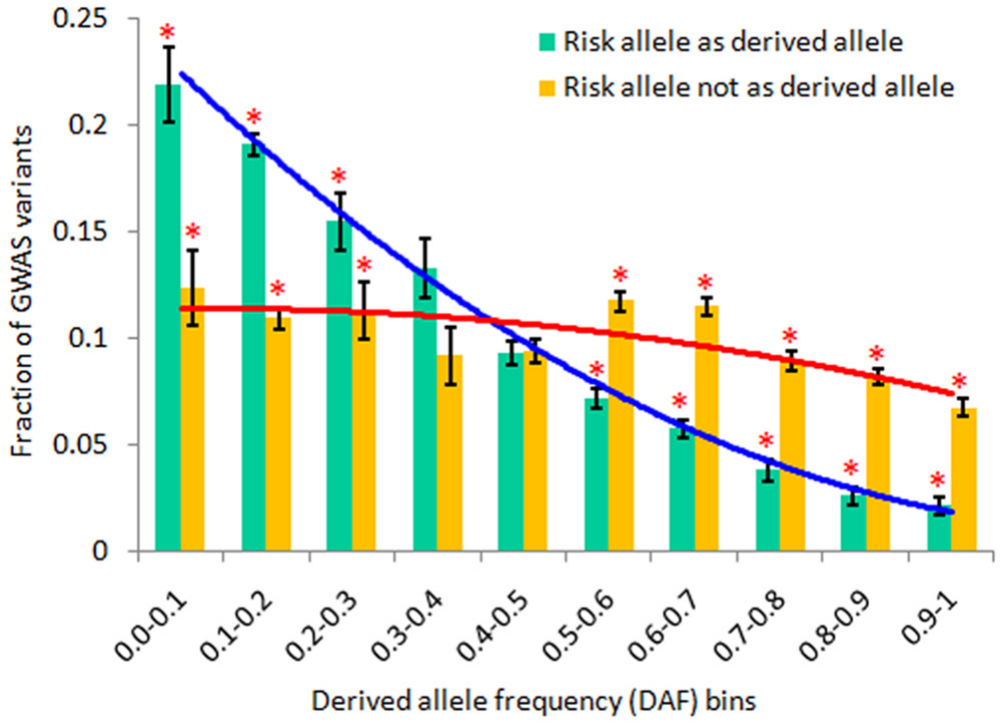
Distribution of GWAS variants for Derived allele frequency (DAF) inferred purifying selection in the human genome. Comparison of combined allele frequency across the worldwide populations in each DAF bin for derived allele as a risk allele (blue line) and as a non-risk allele (red line). Results showed that purifying selection maintains the risk allele at a lower frequency while non-risk allele’s frequency showed nearly similar pattern in all the DAF bins, which could be a consequence of genetic drift in the populations. The astral represents significant differences between both of the datasets in each DAF bins (Fisher exact tests, *P*< 0.05). The error bar represents standard deviation of combined dataset across the populations.

Genome-wide Association Studies (GWAS) have enabled the identification of thousands of diseases or trait-associated variants, with the majority of these variants falling within the noncoding regions [26]. Here we attempted to investigate whether NCS variants identified by GWAS underlie natural selection and have functional potentials. First, we curated GWAS variants based on the information given in the GWAS catalogue into external replicated, internal replicated and un-replicated categories. A total of 5,057 variants were mapped to our data (S20A Fig). Out of the 5,057 variants, 46% were internal replicated, 7% external replicated, and 47% were un-replicated. Again, element-wise distribution showed that nearly 1% of variants mapped to the CDS, while the rest of the variants were from noncoding regions (S20B Fig). Further, using risk allele information from GWAS, we identified 1,445 variants in our dataset having known as derived allele state. Out of these variants, 827 variants had derived alleles as risk alleles while in the rest of the GWAS variants, derived alleles were not associated with risk alleles. Our results showed that a major fraction of the GWAS variants where risk allele was derived allele fell within the lower DAF bin < 0.5, while in the second category wherein the risk allele was not a derived allele, most of the GWAS variants were within intermediate or higher DAF bins in worldwide continental populations (Fig 6, S21A and S21B Figs). The high number of frequency of derived allele as a risk allele for various human diseases in lower DAF bin in worldwide populations indicated the negative selection pressure acting on deleterious variants in the human genome. On the contrary, the spectrum of the non-risk derived allele suggested that natural selection or genetic drift had maintained such variants nearly uniform in the populations. However, we could not exclude the possibility of the discovery bias of case/control studies which favourthe detection of low-frequent alleles that increase disease risk because of the over-sampling of cases.

### Footprints of positive selection in diseases associated with NCS

The roles of NCS elements in health and disease have been functionally observed by several studies, such as those studies on lincRNAs and miRNAs [53–55]. The 5′UTR region of a gene is enriched for regulatory elements such as promoter activity, which regulates nearby genes in a *cis*-manner, while 3′UTRs are sites for miRNA binding and regulation of gene expression [56]. To examine natural selection on the NCS variants associated with diseases, here we classified NCS variants into three categories based on statistics of: i) DAF > 0.5, ii) *F*_ST_ > 0.3, and iii) |iHS| > 2. Variants that fulfilled any two categories were annotated near to the 5 kb flanking or overlapping genes. We also looked for such variants with regulatory markers that fell within class 1 and class 2 of RegulomeDB (because these categories are highly enriched in functional elements). Based on this, we identified 9,895 variants from 5,253 genomic regions (CDS and NCS pooled) from all the studied populations. In the genome, element-based distribution was as follows, 3′UTR (416), 5′UTR (292), intron (8,283), CDS (386), CNC (212), pseudogene (93), lincRNA (103), miRNA (4), miscRNA (2), rRNA (1), snRNA (1), snoRNA (2), and tRNA (1). We observed a small fraction of the genome with putative selection events on protein coding sequences while the majority of the positive selection events were contributed by NCS sequences in the genome, consistent with previous studies [57]. Furthermore, we observed that a small set of NCS variants were highly enriched for regulatory markers associated with diseases or phenotypes in GWAS and were positive for a signature of selection for NCS regions in worldwide human population, as shown in S22 Fig Examples of these are: an intronic variant rs4763879 of *CD69* gene associated with Type I diabetes in Asian and European populations [58], an intronic variant rs2618476 of *BLK* gene associated with Systemic lupus erythematosus (SLE) in Asian populations [59], *APOE* associated with Alzheimer's disease (AD) in African populations [60], *SC4MOL* associated with insulin-related traits [61], IBD5 associated with Crohn's disease [62], *RAF1* associated with cardiac hypertrophy [63], and *DNAH10* associated with visceral adipose tissue adjusted for body mass index in Asian populations [64]. These selected variants could be potential causal factors in human diseases or phenotypic differences and thus are important in medical genetics.

### Gene enrichment analysis of NCS eQTLs showing a signature of positive selection

We further investigated the gene enrichment of eQTLs that showed a signature of positive selection and were associated with differentially expressed genes in European (CEU) populations. For this, we obtained the eQTL data from eQTL Browser for the NCS variants that showed positive selection based on cutoff threshold discussed before. Next, we manually curated the mapped data for expressed genes that were present in European populations, and identified 166 differentially expressed genes associated with positive selection in Europeans. These identified genes were further used for multiple gene feature enrichment analysis using ToppCluster. At a false discovery rate (FDR) of 5%, we observed a significant enrichment of *SLC22A4*, *SLC22A5*, and *P4HA2* genes for Crohn’s disease and inflammatory bowel disease (IBD) (S23A–S23C Figs). In addition, we also observed the enrichment of nuclear encoded mitochondrial genes that influence AIDS and are of the chr16q22 cytoband region. In our dataset, five SNPs (rs2631360, rs17622208, rs11748193, rs12521868, and rs17622656) were *cis*-eQTL variants for genes *SLC22A4* (*OCTN1*), *SLC22A5*(*OCTN2*), and *P4HA2* in CEU populations. All these eQTL variants showed higher derived allele frequency in EUR and AMR populations except for rs2631360, which was also present in high frequency in AFR populations. One of the variants, rs12521868, located in the intron of *C5orf56* has been previously reported in GWAS as a Crohn’s disease (CD) susceptibility locus in European populations [65]. We used HaploReg v2 tool to annotate the regulatory functions of these noncoding variants. As shown in S23C Fig, the log of odds of PWM score is greatly affected by the change in allele states from ancestral to derived allele of regulatory motifs, and this might impact gene regulation. Based on IBD endemicity related study, it has been known that this disease is highly prevalent in European and American populations [62, 66]. One of the positively selected variant, rs12521868, has been previously identified as IBD-associated and encompasses foxp3 regulatory motif that interacts with RUNX1 which is involved in the control of T-cell mediated immune responses [65, 67]. Also, RUNX1, an essential hematopoietic transcription factor, is associated with the regulation of *SLC22A4* [68]. It has been known that in Crohn’s disease and ulcerative colitis, patients have activated innate (macrophage, neutrophil) and acquired (T and B cell) immune responses and loss of tolerance to enteric commensal bacteria [69]. Here, our results suggest that selection of these eQTL NCS variants in European populations and alteration of foxp3 motif and subsequent interaction with RUNX1 and *OCTN1* might play important roles in understanding the endemicity of IBD.

## Discussion

In the recent years, contribution of noncoding DNA elements has been greatly appreciated in various important biological functions and diseases. We investigated the functional potentials of noncoding DNA sequences and provided a comprehensive and systematic analysis of NCS elements for detecting natural selection and attempted to show their functional potentials.

In summary, we observed that both natural selection and demographic forces have globally shaped the underlying genetic variations of noncoding and coding DNA sequences in the genome. However, by analyzing genetic variants data of NCS elements in the populations with respect to the genome background, we are able to demonstrate that natural selection forces have profound influence on the NCS elements such as coding sequences, indicating functional constraint. NCS elements such as 5′UTR, 3′UTR, tRNA, intron, CNC, and most of ncRNAs showed strong purifying selection pressure, while pseudogene, intergenic and lincRNA showed weak constraint elements in the genome. These observations were consistent with site frequency spectra (SFS) of neutrality statistics as well as allele frequency spectrum such as MAF and DAF. Furthermore, lines of evidence of enrichment of low population differentiation variants in NCS regions and enrichment of variants with low *F*_ST_ prevalent in rare allele frequency support our results from SFS based analysis. In addition, we observed that a large proportion of non-conserved noncoding variants have rare derived allele frequency in the genome, which suggested that NCS regions of these variants underwent purifying selection in recent history of modern human. Furthermore, our analysis showed that the prevalence of the risk alleles identified by GWAS as derived alleles in the humans are under strong negative selection and restricted to lower allele frequency in worldwide populations. Interestingly, tRNA exhibited strong signature of purifying selection that is more pronounced than CDS elements, though tRNA showed the higher nucleotide diversity among the studied elements. We concluded that purifying selection maintains functionality of tRNA gene for codon usage bias and prevents it from mis-incorporation of amino acids in protein synthesis, while the high diversity of tRNA sequences resulted from their various functions as tRNA isodecoders other than translation [31, 49, 70]. Our analysis revealed that NCS elements are much more enriched for high differentiation variants than CDS regions. However, the intergenic region is significantly depleted for such variants. Furthermore, 3–24% of NCS variants had higher iHS scores in the four continental populations with the highest in African populations (24%) compared to less than 1% of CDS variants in the studied populations, indicating that NCS variants are more common than CDS to be underlying positive selection. This observation was further supported by the enrichment of NCS variants with high *F*_ST_ and high iHS score in the genome. However, it is also possible that the hitchhiking effect of positively selected variants in functionally more important regions might affect the occurrence of the variants of flanking regions especially those with low recombination [38, 71]. Our analysis showed that NCS elements are highly enriched for regulatory markers in the genome,indicative of various regulatory roles in human populations. Interestingly, our functional annotation revealed that 5′UTR regions are highly enriched for regulatory markers such as eQTLs, DNase I, and TF binding motifs and indicated that 5′UTR plays an important role in gene regulation [29]. We also observed that some of the positively selected NCS variants, which were previously identified as disease-associated by GWAS, were highly enriched for various regulatory markers that distributed in a cell type-specific manner. These results indicated that the molecular mechanisms involved vary according to cell type and disease and are potentially informative in understanding the etiology of diseases in populations.

The SFS based neutrality tests with sequencing data provide robust estimation of deviation from the neutral model of genomic elements influenced by demographic as well as evolutionary forces. However, this estimation was dependent on data such as SNP density, size of element, and length of window taken for analysis that confounding effect was very difficult to control in dealing with elements of various lengths [72]. In this analysis, we tried to overcome this effect by concatenating genomic elements with fixed length of window to calculate the neutrality tests, and we incorporated a bootstrapping approach to generate equal number of samples from each element for further analyses. In addition, to detect signatures of natural selection, a background was usually established for comparison based on the entire genome or pseudogene, as these are supposed to be nonfunctional. However, recent evidence has suggested that some of these pseudogenes gain functionality in the form of ncRNAs such as lincRNA or by becoming transcriptionally active which may affect the analysis [30, 54]. Here, we used the genome as a background for comparison as the majority of the genome is neutrally evolving and any deviation from the genome is suggestive of selection pressure on a particular genomic element. We also observed that different demographic forces globally shaped coding as well as noncoding DNA sequences across the continental populations and that may contribute to the uneven pattern of enrichment of genetic variants of elements between the populations. This is in spite of the majority of NCS elements showing signature of purifying selection in the genome. In addition, recombination rates of genomic regions and linkage disequilibrium (LD) are also contributing factors that may elevate the frequency of variants especially in positively selected variants as a result of genetic hitchhiking in low recombination regions [71].

One of the major questions in population and evolutionary genetics has been what fraction of the genome has undergone purifying selection because addressing this question can reveal potential functionality [20, 73]. A recent study by Lucas and Manolis on non-conserved regulatory elements has identified additional 4% of the genome subject to lineage specific purifying selection, though this percentage is very small compared to ENCODE estimate of functional markers in diverse cell lines [22]. However, it is challenging to estimate the fraction of the genome underlying selection by correcting for reference background in empirical data as this was limited by arbitrary cutoff of tail of empirical distribution when any statistics is applied. Another aspect that may contribute to different selection pressures within a genomic element is different motifs and their functional importance in regulation [17, 22, 52, 74]. In our analysis, we used the entire pre-miRNA sequences to ease the complexity and the majority of statistics showed moderate selection pressure compared to strong selection pressure on seed regions in earlier studies [25, 75]. In addition, computational limitation in the calculation of summary statistics is one of the major challenges when dealing with large number of sequences in huge data sets like the 1000 Genomes.

Variants that showed signal of positive selection might play important rolesin understanding the prevalence of diseases or traits in the populations [57, 76–78]. We identified eQTL loci from NCS region that showed high *F*_ST_ and iHS differences in the European populations, and our gene cluster enrichment analysis observed enrichment of immune-related genes, *SLC22A4*, *SLC22A5*, *P4HA2* in Europeans that showed association with IBD and diet-related phenotypes [62, 66]. This gene-clustering based approach allowed us to prioritize positively selected variants for further case control studies and dissect the role of such variants in diseases. Furthermore, population-specific positive selection of miRNAs contributes to the adaptation to specific environment and is potentially important in biological functions [25, 79]. Based on pairwise *F*_ST_ differences (*F*_ST_ > 0.3), 65 variants from 59 miRNAs showed continental specific differences (S3 Table). Out of 65 variants, rs2427556 of has-miR-941-1 present in chromosome 20, showed high *F*_ST_ differences between Asian and European populations (0.406 for CHB-CEU). Recently, it has been reportedthat mir-941 emerged *de novo* in humans from an evolutionarily volatile tandem repeat sequence, and multiple copies of this miRNA are present in the human genome [80]. We identified two more variants apart from rs2427556, which are present in miR complementary region. These variants are rs4809383, which is present in the stem region of mir-941-1 and rs7320929, which is present in mir-941-3. The allele frequency of rs2427556 was much higher in Asian populations (65% in CHB) than in European populations (15% in CEU), while rs4809383 was nearly the same in worldwide populations (S24A–S24C Figs). Although we have not observed continental specific differences for variants rs7360929 of mir-941-3, we did observe that, after scanning100 kb of the flanking region,the miR-941 region showed selection signatures in the Asian populations (shown in red color) while the flanking regions did not. In addition, we observed a regulatory motif HDAC2 underlying the rs2427556 variant that showed significant differences in PWM score with respect to reference allele versus altered allele for this motif. HDAC2 played a role in the insulin signaling pathway and in synaptic plasticity which was observed in the adult mouse hippocampus [81]. Also, mir-941 targets genes shown to be involved in insulin pathways and neurotransmitter signaling [80]. These evidences indicate that allelic differences in rs2427556 of mir-941-1 and HDAC2 might play important roles in cognitive impairment associated diabetes mellitus, also known as type 3 diabetes and was observed in cases of Alzheimer’s disease [81]. Another example of a selected NCS variant, based on pairwise *F*_ST_ differences between European and African populations,is rs13303010 from *NOC2L* (S25 Fig). *NOC2L* is a novel HDAC-independent inhibitor of histone acetyltransferase (INHAT), which can regulate histone modification [82]. These evidences suggested that NCS elements are important for biological functions and can be looked into further details at molecular and functional levels.

## Conclusions

In conclusion, we demonstrated that NCS in the human genome are globally shaped by purifying selection, indicating their potential functionality. In addition,a significant fraction of NCS variants might play a role in driving differential biological functions and phenotypes in worldwide human populations, as a result of positive selection. However, further verification and especially experimental studies are necessary to confirm our observations and results.

## Supporting Information

S1 Fig Box plot of length distribution for elements studied in the human genome. The highest range of length was observed for intron while the lowest was observed for tRNA genes. In the inset, smaller elements were re-plotted for visualization. The length on Y-axis is in log of base 10 of base-pair (bp).
(PDF)

S2 Fig SNP density plot of studied elements. tRNA genes exhibited the highest density and pseudogenes exhibited the lowest density in all the studied elements.
(PDF)

S3 Fig Human demography of modern human populations. Genome-wide Tajima’s *D* density plot revealed demography events of African and non-African populations. A skew in the negative tail of *D* for non-African populations is indicative of population expansion while a skew in the heavy tail of positive *D* is indicative of population bottleneck of human populations in the past. Non-African populations underwent severe bottleneck.
(PDF)

S4 Fig Beanplot representation of pairwise nucleotide diversity (π) in four populations. The middle horizontal bar represents mean π of genomic elements.
(PDF)

S5 Fig PCA plot based on resampled SNPs for each element. For PCA analysis, resampled data of 870 markers were used from each element because rRNA has only 870 markers. The blue color represents European, red for Asian, black for African, and green for American populations. Data on American populations showed more closeness to European populations.
(PDF)

S6 Fig Individual-based clustering using STRUCTURE at K = 3. At K = 3, individuals were clustered into three groups (African, Asian, and European ancestry). American populations showed a different level of admixture with Asian and African populations. Some of the NCs elements displayed heterogeneity at the structural level and these elements exhibited more heterozygosity than other elements (S5 Fig).
(PDF)

S7 Fig Estimation of observed heterozygosity (*H*o) of the various genomic elements. A higher *H*o was observed for tRNA, whereas the smallest *H*o value was observed for CDS elements.
(PDF)

S8 Fig Minor allele frequency spectrum of all the studied elements in all the 1000 Genomes Phase I populations. (PDF)

S9 Fig Minor allele frequency (MAF) < 0.05 of studied elements in (A) YRI, (B) CHB, and (C) JPT populations. Compared to the genome-wide background, intron, CDS, 5′UTR, 3′UTR, tRNA, and CNC had significantly enriched rare variants; pseudogene and intergenic element showed significant depletion; ncRNAs exhibited a high number of low MAF variants.
(PDF)

S10 Fig Derived allele frequency < 0.05 spectrum across all studied populations for all studied elements. African populations had the highest fraction of DAF < 0.05 compared to Asian and European populations.
(PDF)

S11 Fig Derived allele frequency (DAF) < 0.05 of studied elements in (A) YRI, (B) CHB, and (C) JPT populations. Compared to the genome-wide background, intron, CDS, 5′UTR, 3′UTR, tRNA, and CNC had significantly enriched rare variants; pseudogene and intergenic element showed significant depletion; ncRNAs element showed population-specific significant enrichment for low DAF variants.
(PDF)

S12 Fig Global *F*_ST_ values for various genomic elements in the human genome. tRNAs showed the lowest differentiation and miscRNA showed the highest differentiation.
(PDF)

S13 Fig Mean pair-wise *F*_ST_ differences among the three continental populations for various genomic elements. In the genome, miscRNA showed the highest *F*_ST_ differentiation while tRNAs showed the lowest differentiation between any continental populations. Error bar indicates the standard deviation of mean of *F*_ST_.
(PDF)

S14 Fig Fraction of global *F*_ST_ variants < 0.05 in the various genomic elements. The number of low *F*_ST_ variants is significantly high for CDS, Intron, 3′UTR, tRNA, and CNC but low for intergenic elements when compared to the genome-wide background. miscRNA, snoRNA, snRNA, and rRNA also showed a high number of such variants but the values obtained were not significant (χ^2^ test, *P* < 0.05). Also, 5′UTR showed less number of these variants but this difference in value from the genome-wide background was not significant. The dashed line indicates genome-wide threshold.
(PDF)

S15 Fig (A) Conserved and (B) non-conserved variants with low DAF ≤ 0.05. (PDF)

S16 Fig The fraction of global *F*_ST_ variants > 0.4 in the various genomic elements. The number of high *F*_ST_ variants is significantly higher for intron than that for the genome-wide background, whereas that for pseudogenes was marginally significant compared to the genome-wide background. However, CDS, tRNA, and CNC showed significant depletion for such variants.
(PDF)

S17 Fig Enrichment of high *F*_ST_ variants in intermediate MAF bins. In this analysis, the number of high *F*_ST_ variants was higher in NCS elements in different high MAF bins than in the genome-wide background when compared according to their respective bins (A) at > 0.4 (B) at > 0.6. (Red filled blocks represent significant differences: χ^2^ test, *P*< 0.05 used for all comparison, NS: not significant).
(PDF)

S18 Fig Long range haplotype-based analysis showed enrichment of NCS elements. In our dataset, |iHS| score ≥ 2 variants showed significant enrichment in NCS sequences but depletion for CDS in the genome (χ^2^ test, *P* < 0.05) in all the populations (A). Also, with high |iHS| > 2 and high *F*_ST_ > 0.3 variants, we detected a significantly enrichment for NCS sequences compared to CDS (χ^2^ test, *P* < 0.05) in all the populations (B).
(PDF)

S19 Fig Small ncRNAs were subject to selection based on integrated haplotype score. A significant number of ncRNAs variants were positive for the selection indicator, the |iHS| score in the human genome, and thus could have been a potential target for local adaptations.
(PDF)

S20 Fig GWAS revealed variants distribution in dataset. (A) Classification of validated associated variants from GWAS used in this analysis. (B) Distribution of GWAS variants in the various genomic elements.
(PDF)

S21 Fig Derived allele frequency (DAF) spectrum of GWAS-associated variants in worldwide human populations under purifying selection. (A) Derived alleles which are risk alleles based on GWAS studies had frequency distribution restricted to lower DAF bins, (B) whereas derived alleles which are not risk alleles showed higher frequency in higher DAF bins. The combined data represents derived allele frequency across all the populations, and the error bar represents standard deviation.
(PDF)

S22 Fig Positively selected variants associated with GWAS and regulatory markers. These variants are highly enriched for underlying regulatory markers for methylation, acetylation, DNase I, transcription factor binding sites, and protein bound motifs. These markers are represented accordingly in the three cell types. The differences in the patterns might be indicative of their roles in physiology and pathophysiology.
(PDF)

S23 Fig Gene set multi-feature enrichment (ToppCluster) in CEU of positively selected eQTL variants and their associated genes identified from eQTL browse and network drowns using Cytoscape software (A), Frequency of alternate alleles in continental populations (B), Log of position weight matrix (PWM) score for regulatory motifs for variants obtained from HaploRegV2 (C).
(PDF)

S24 Fig Positive selection signature in miR-941 of Asian populations. We identified rs2427556, located in the miR complementary region of miR-941-1 in this cluster (shown by dashed box in C), to be positive for the signature of selection based on *F*_ST_ differences. (A) The moving average trend line shows the differences between CEU and CHB populations, and rs2427556 is shown by a black arrow. Two other variants were detected: rs4809383 is present in the stem region of mir-941-1 and rs7320929 is present in mir-941-3. (B) The allele frequency of rs2427556 was higher in Asian populations than in European populations, while rs4809383 has nearly the same frequency in worldwide populations. (C) The lower panel shows the UCSC genome browser view for the miR-941 cluster and iHS score. The X-axis shows the base position on chromosome 20 of the variants.
(PDF)

S25 Fig UCSC Genome Browser view of an NCS variant. The SNP rs13303010, (marked in yellow) in an intron of *NOC2L*, was positive for a signature of selection that overlaps with several regulatory elements. Based on the top 1% high *F*_*ST*_ > 0.3 cutoff, this variant showed differences between European and African populations. *NOC2L* is a novel HDAC-independent inhibitor of histone acetyltransferase (INHAT). Sequence variation in these TFs due to this SNP and gene expression by these TFs in the genome might perturb diverse biological functions in the regulatory network in a cell type-specific manner.
(PDF)

S1 Table Single nucleotide polymorphism (SNPs) distribution of the various genomic elements for different chromosomes from the 1000 Genomes Phase I project. (XLS)

S2 Table Details of site frequency based test of neutrality in this study. The data points presented in bold showed statistical significance compared to genome-wide background based on permutation tests (*P*< 0.05).
(XLSX)

S3 Table Highly differentiated miRNAs among human populations. (XLS)
