# Supplementary figures and images for: Natural Selection and Functional Potentials of Human Noncoding Elements Revealed by Analysis of Next Generation Sequencing Data
Source: PLoS One. 2015 Jun 8;10(6):e0129023. doi: 10.1371/journal.pone.0129023 (PMC4460046; doi:10.1371/journal.pone.0129023)

logarithm of length to base 10

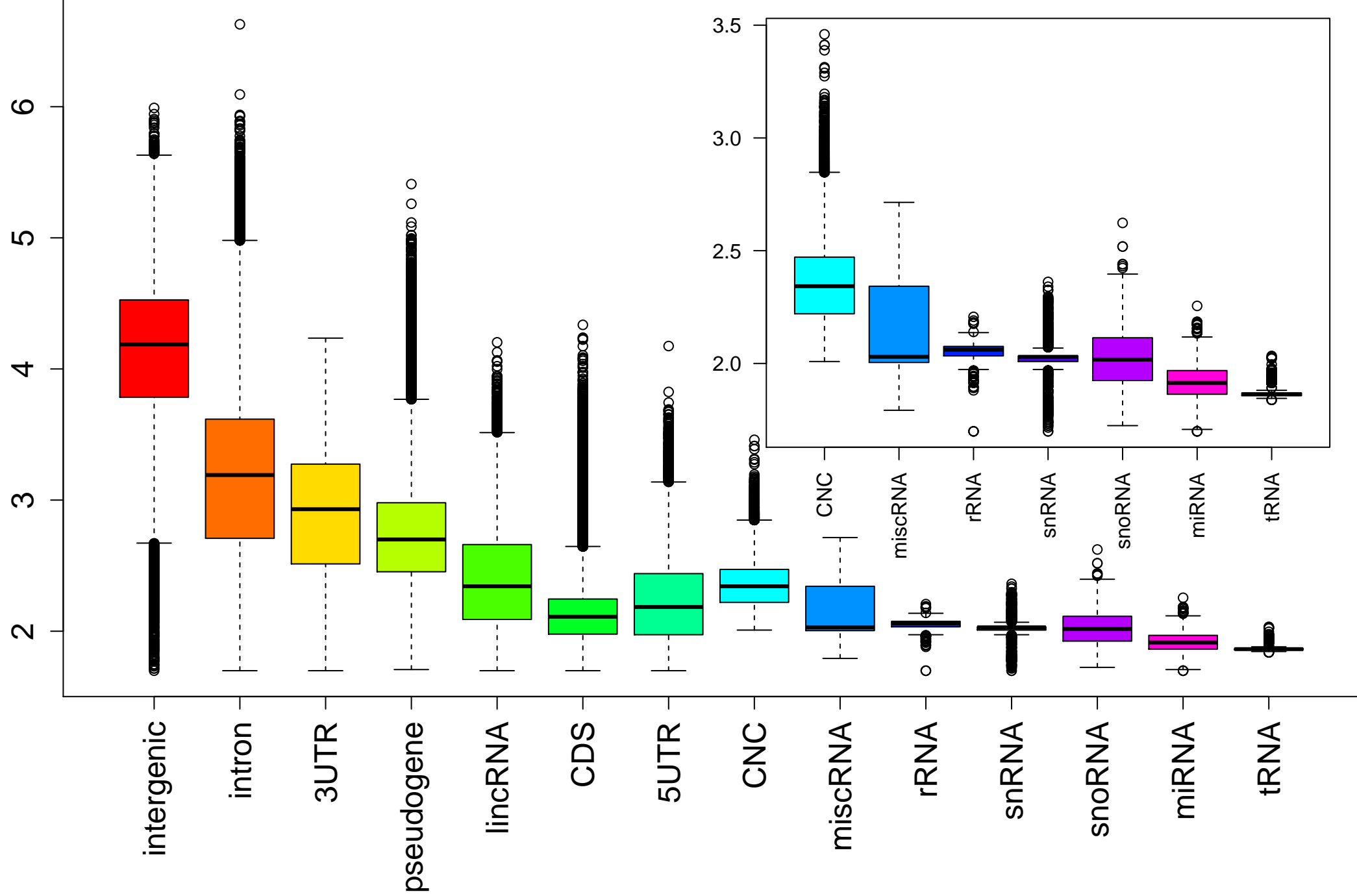

Supplement: S1 Fig — The highest range of length was observed for intron while the lowest was observed for tRNA genes. In the inset, smaller elements were re-plotted for visualization. The length on Y-axis is in log of base 10 of base-pair (bp). (PDF) [file pone.0129023.s001.pdf]

# SNP density(per Kbp)

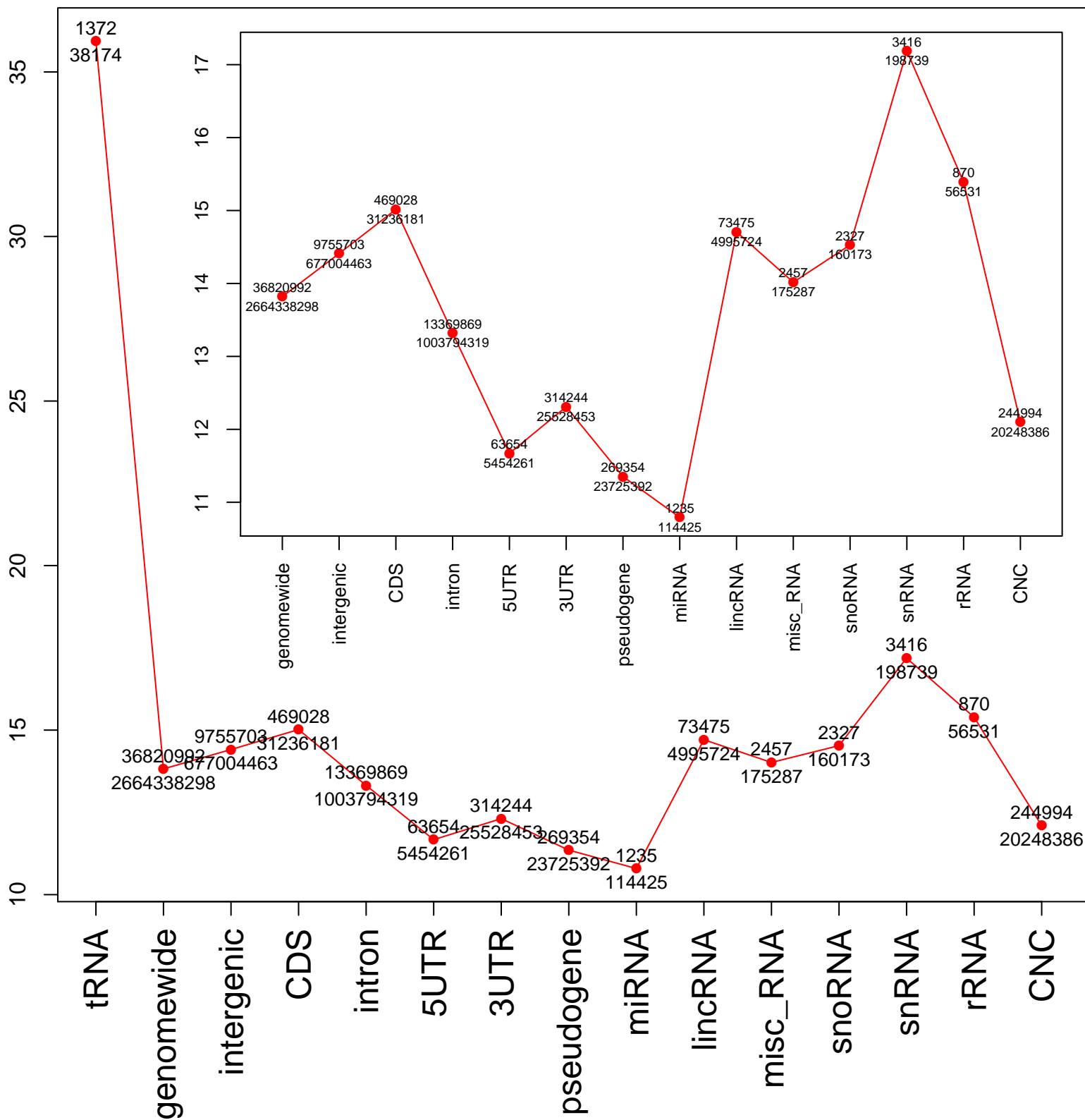

Supplement: S2 Fig — tRNA genes exhibited the highest density and pseudogenes exhibited the lowest density in all the studied elements. (PDF) [file pone.0129023.s002.pdf]

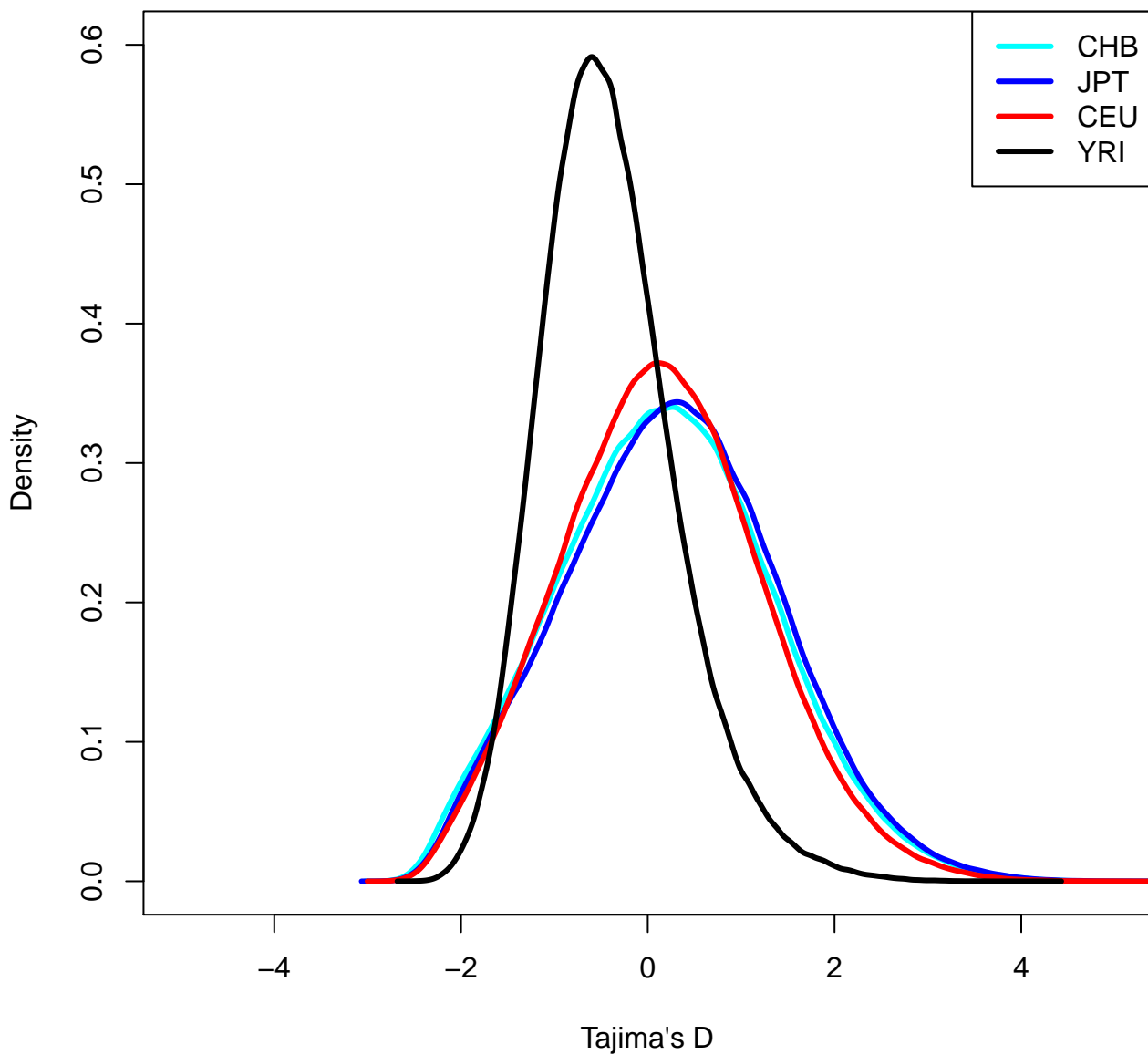

Supplement: S3 Fig — Genome-wide Tajima’s D density plot revealed demography events of African and non-African populations. A skew in the negative tail of D for non-African populations is indicative of population expansion while a skew in the heavy tail of positive D is indicative of population bottleneck of human populations in the past. Non-African populations underwent severe bottleneck. (PDF) [file pone.0129023.s003.pdf]

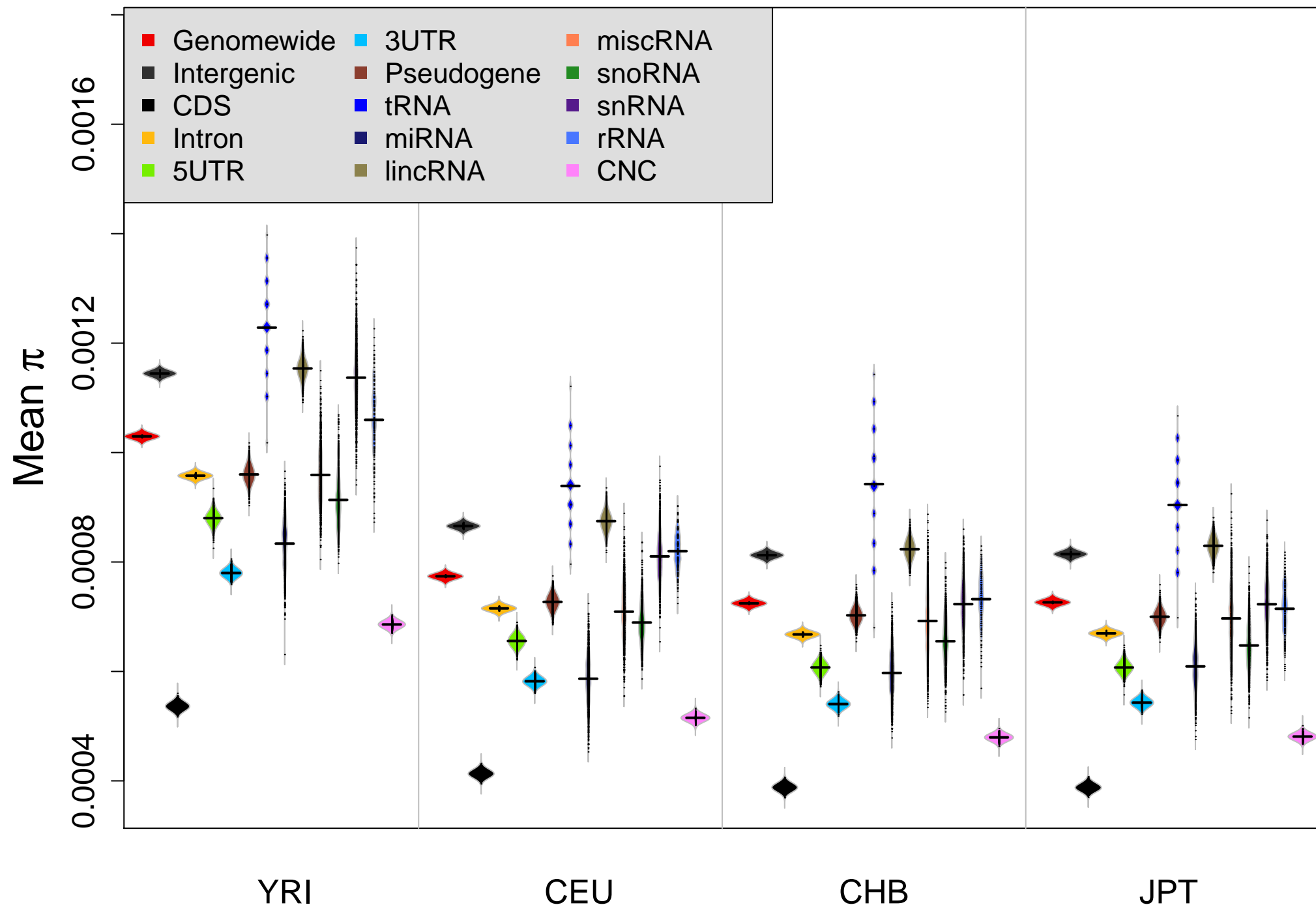

Supplement: S4 Fig — The middle horizontal bar represents mean π of genomic elements. (PDF) [file pone.0129023.s004.pdf]

# K = 3

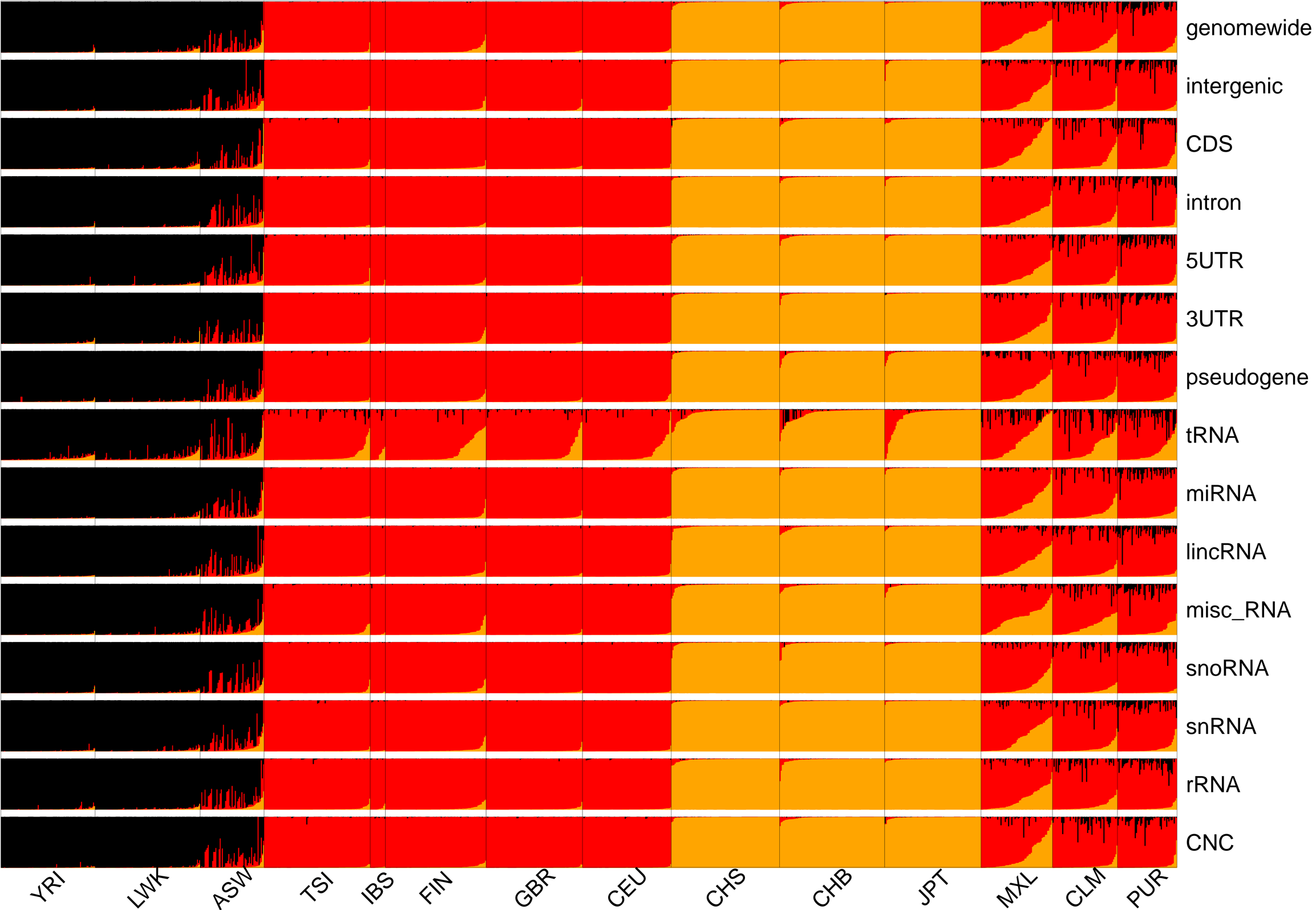

$$K = 4$$
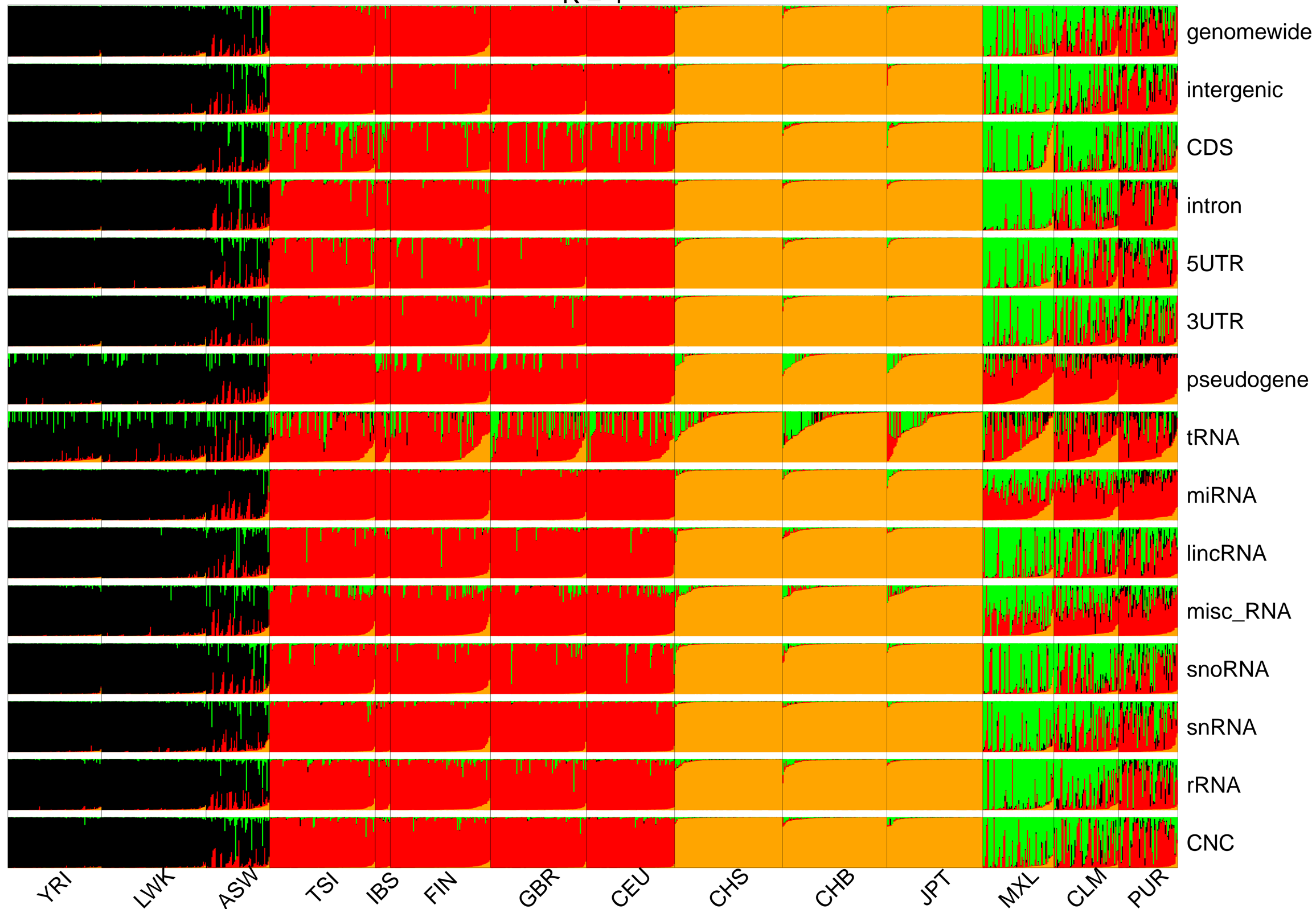

# K = 5

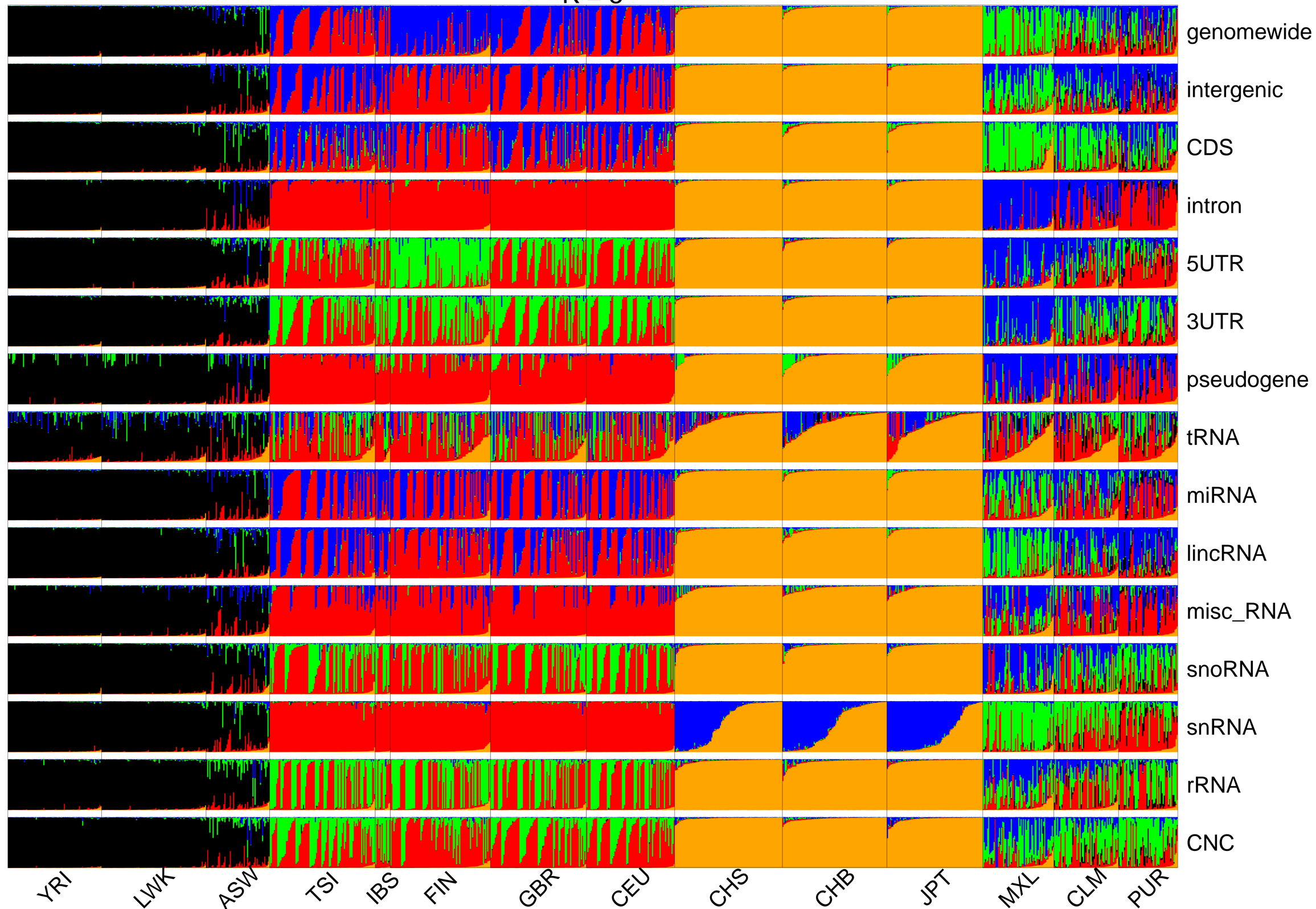

K = 6

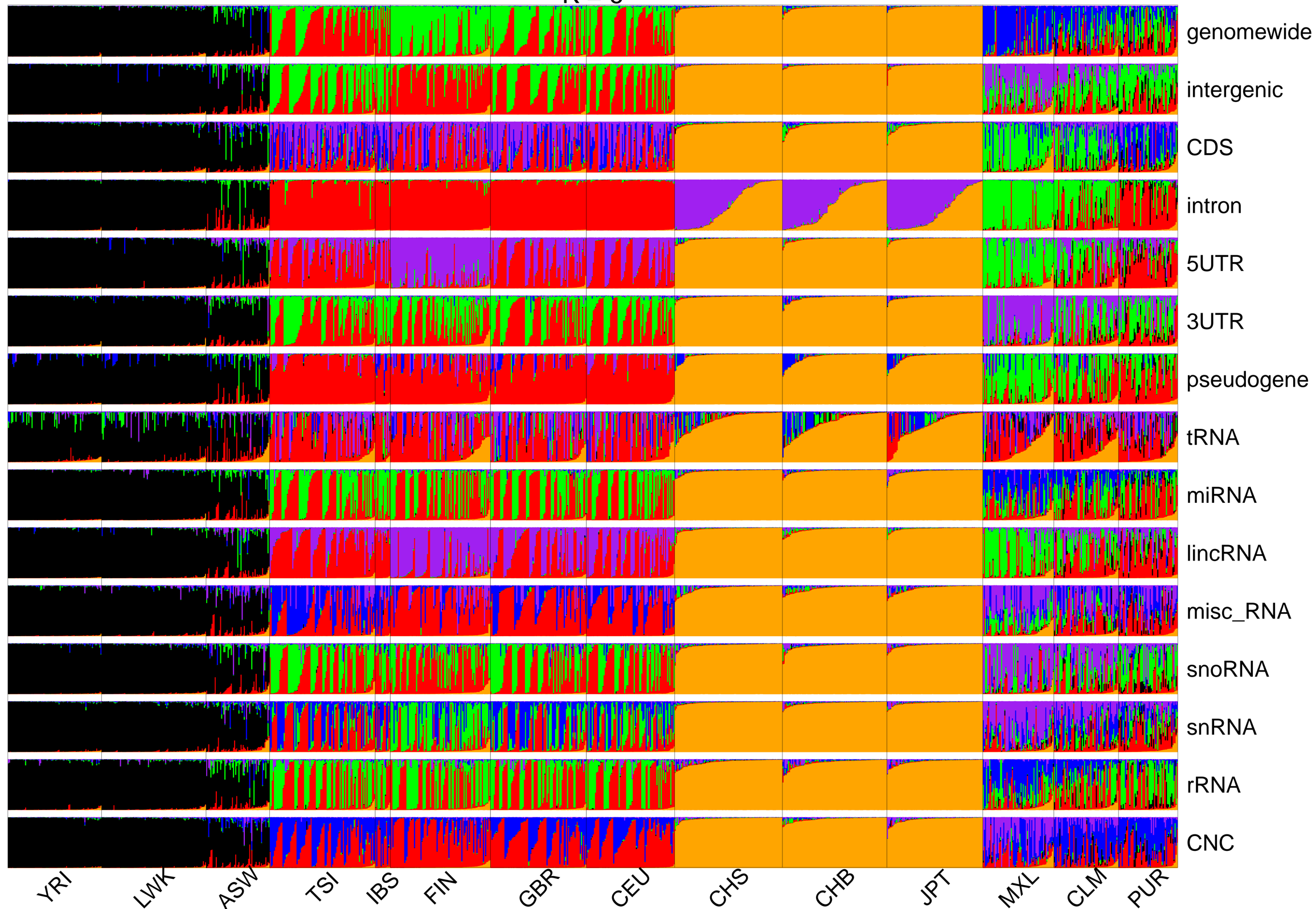

$$K = 7$$
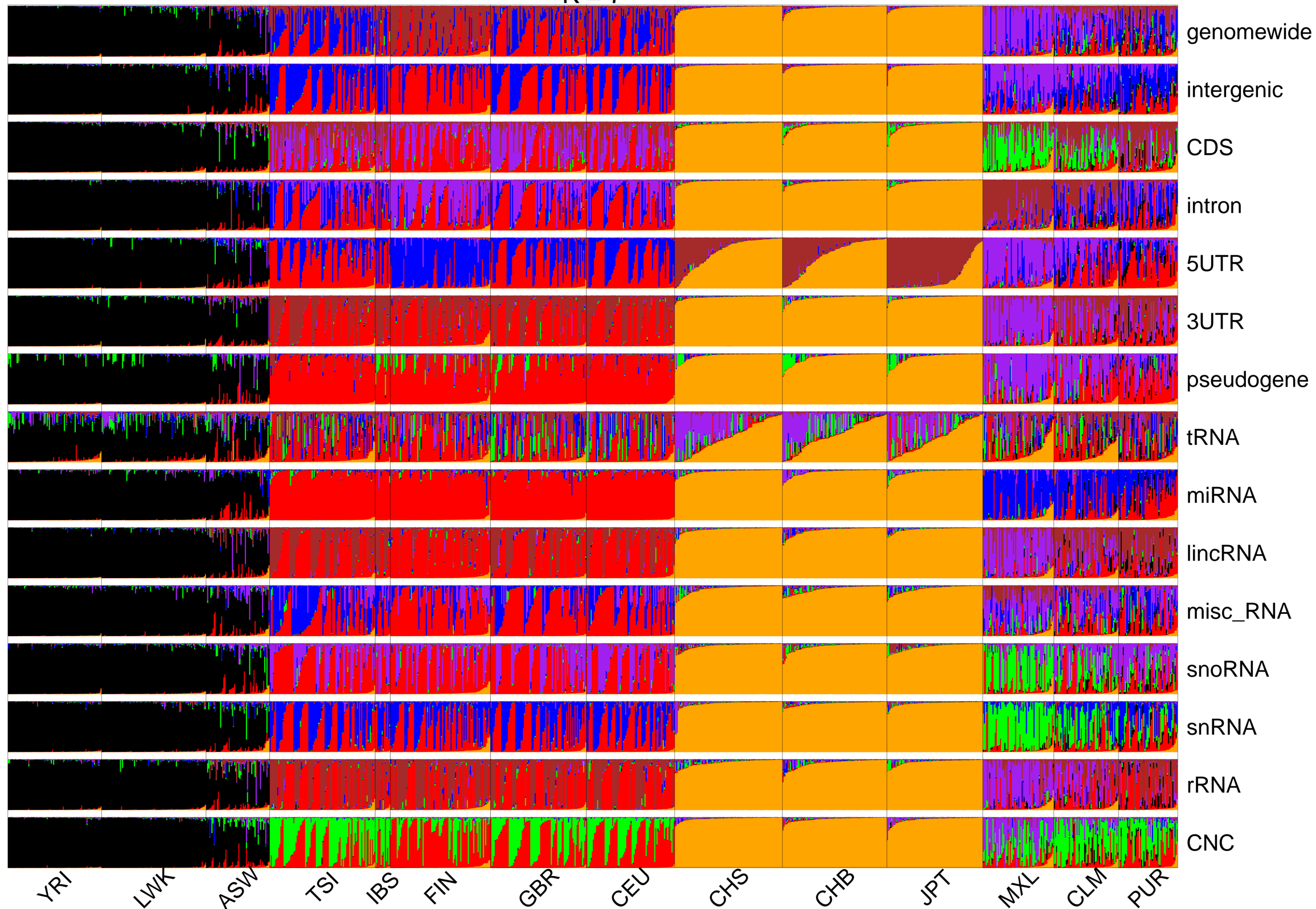

$$K = 8$$
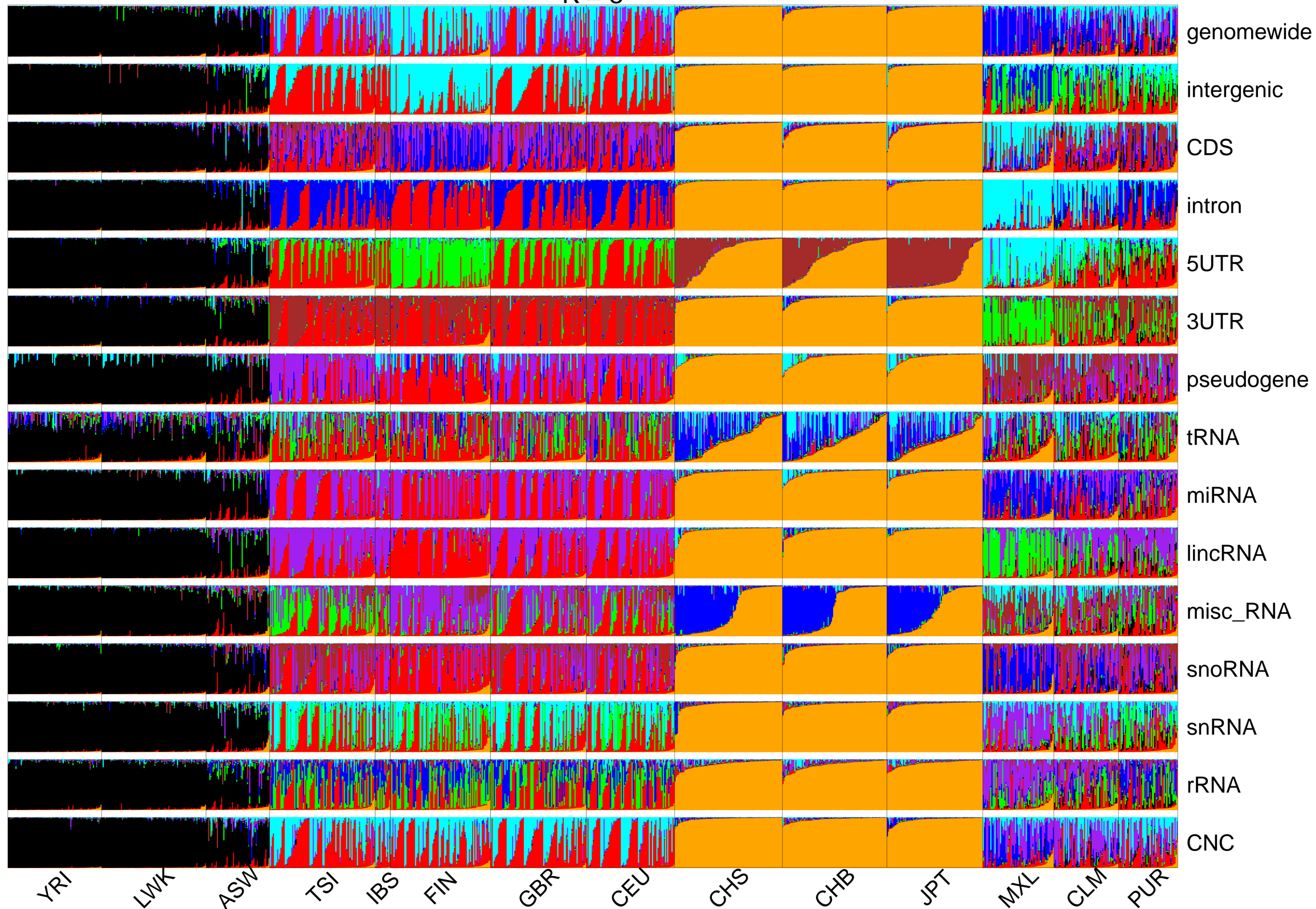

Supplement: S6 Fig — At K = 3, individuals were clustered into three groups (African, Asian, and European ancestry). American populations showed a different level of admixture with Asian and African populations. Some of the NCs elements displayed heterogeneity at the structural level and these elements exhibited more heterozygosity than other elements (S5 Fig). (PDF) [file pone.0129023.s006.pdf]

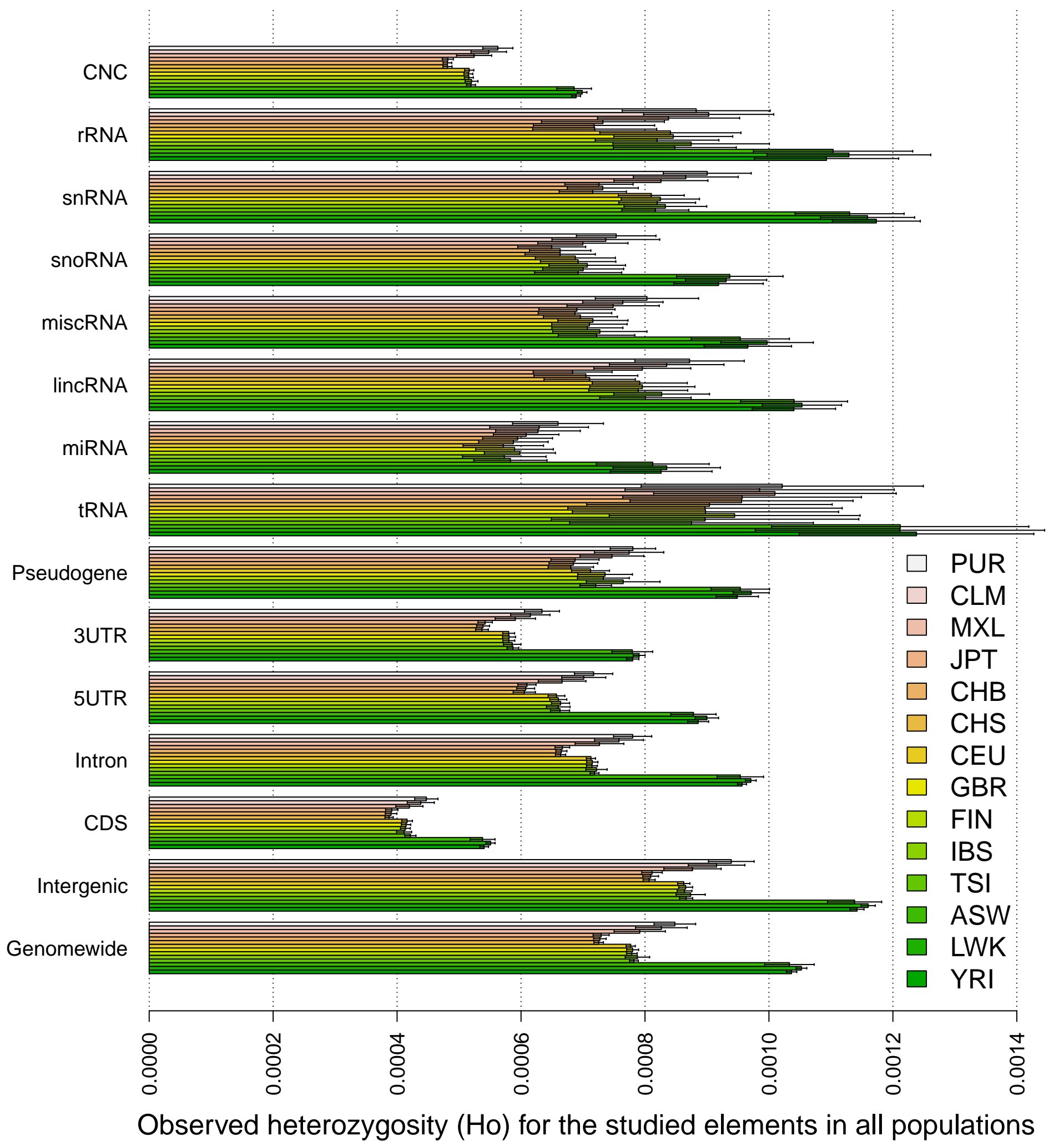

Supplement: S7 Fig — A higher Ho was observed for tRNA, whereas the smallest Ho value was observed for CDS elements. (PDF) [file pone.0129023.s007.pdf]

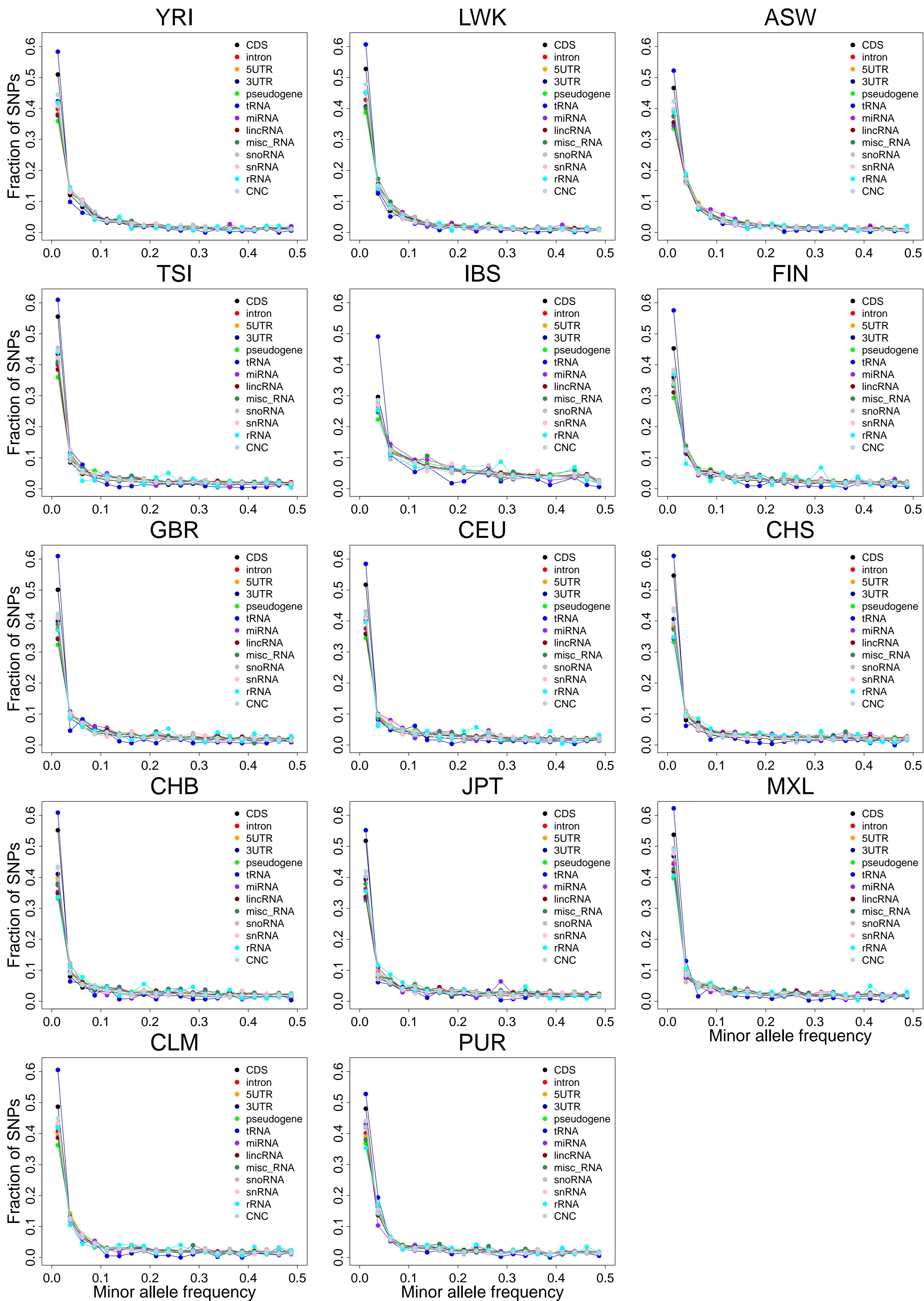

Supplement: S8 Fig — (PDF) [file pone.0129023.s008.pdf]

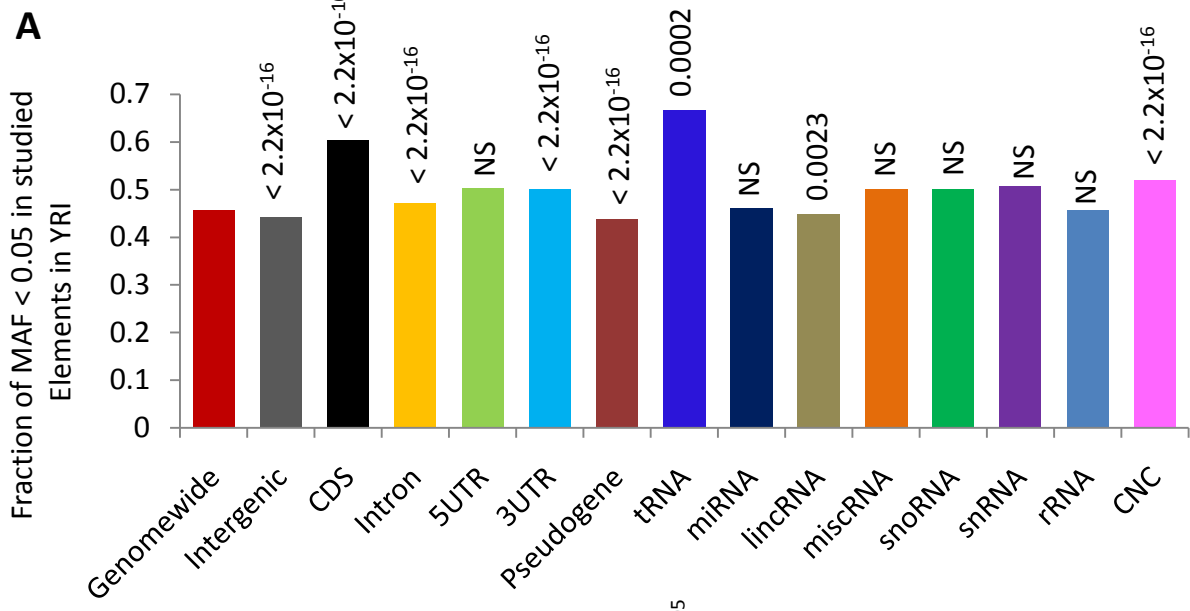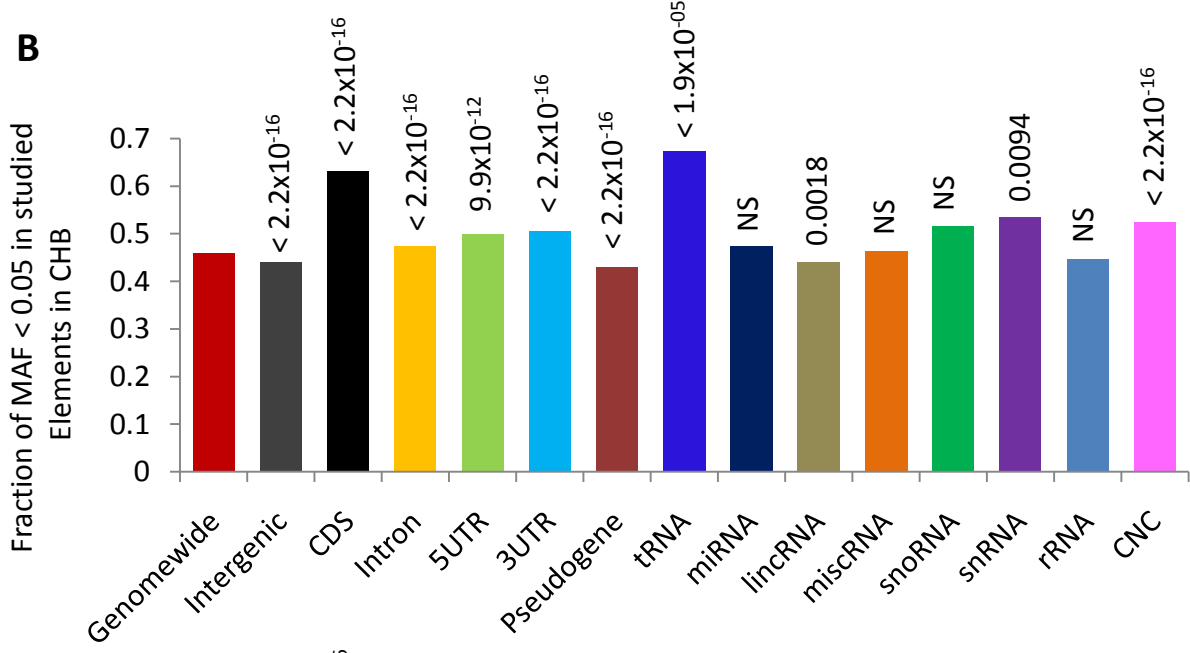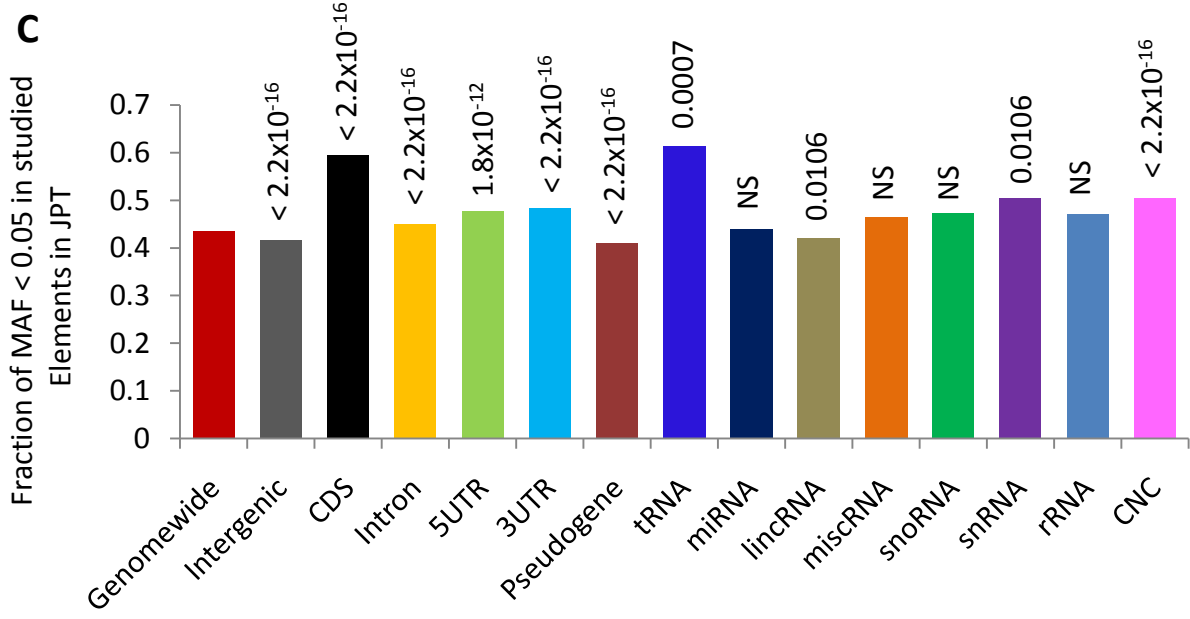

Supplement: S9 Fig — Compared to the genome-wide background, intron, CDS, 5′UTR, 3′UTR, tRNA, and CNC had significantly enriched rare variants; pseudogene and intergenic element showed significant depletion; ncRNAs exhibited a high number of low MAF variants. (PDF) [file pone.0129023.s009.pdf]

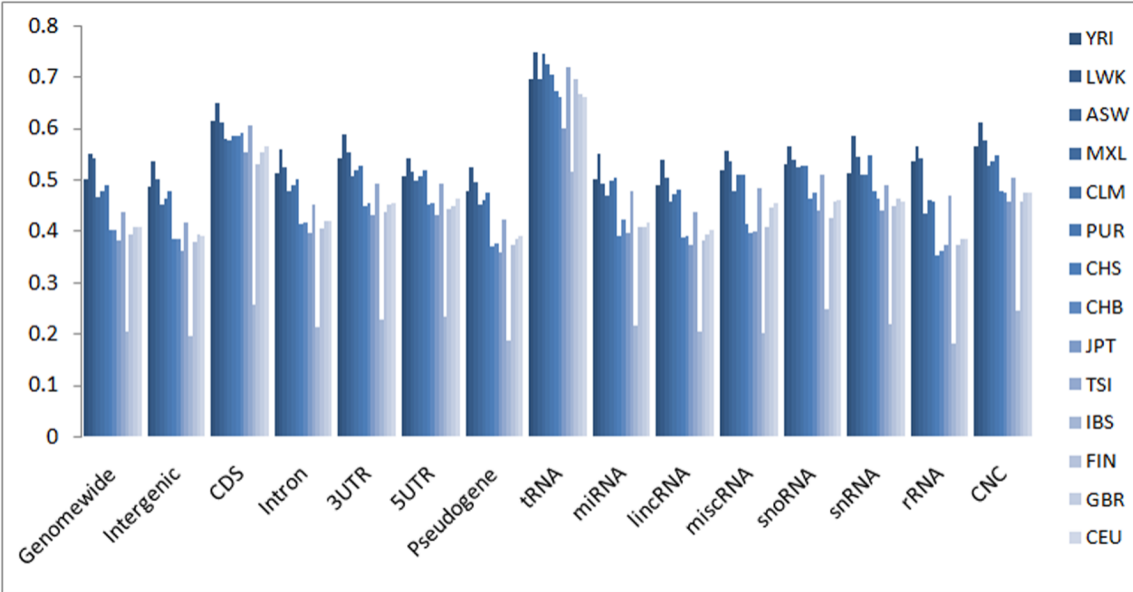

Supplement: S10 Fig — African populations had the highest fraction of DAF < 0.05 compared to Asian and European populations. (PDF) [file pone.0129023.s010.pdf]

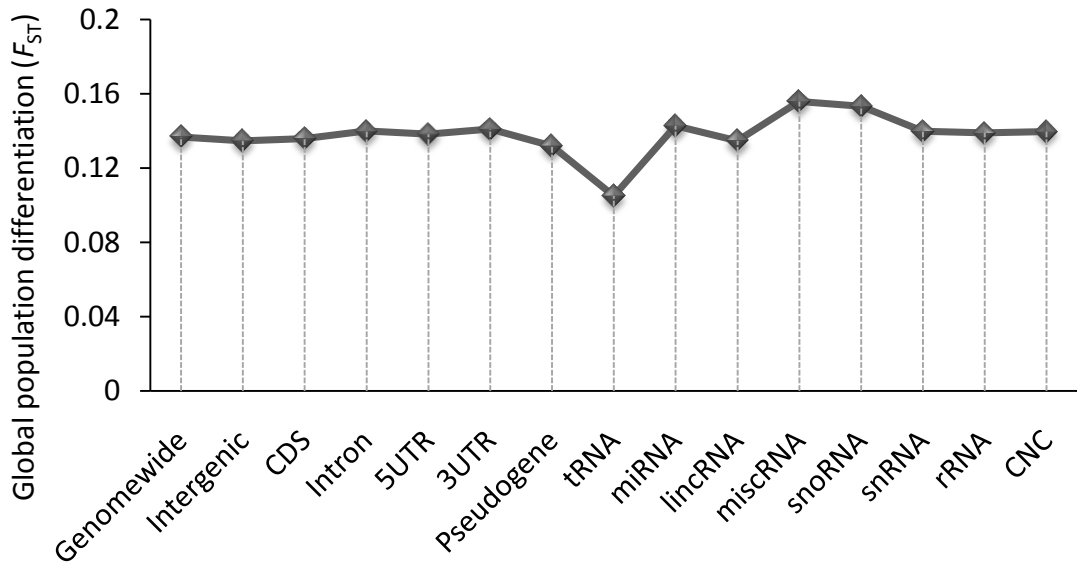

Supplement: S12 Fig — tRNAs showed the lowest differentiation and miscRNA showed the highest differentiation. (PDF) [file pone.0129023.s012.pdf]

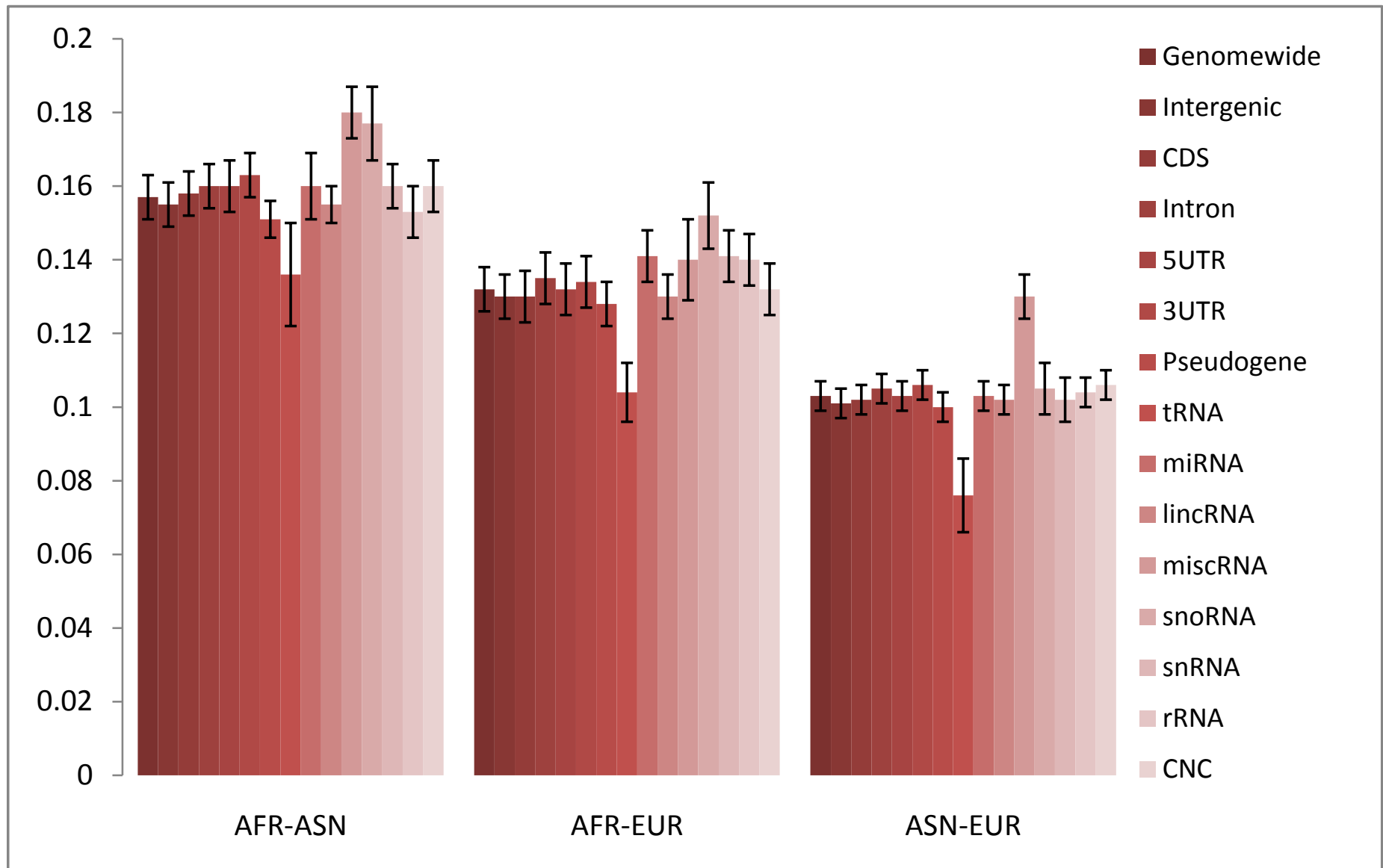

Supplement: S13 Fig — In the genome, miscRNA showed the highest F ST differentiation while tRNAs showed the lowest differentiation between any continental populations. Error bar indicates the standard deviation of mean of F ST. (PDF) [file pone.0129023.s013.pdf]

Fraction of variants having global  
 $F_{ST} < 0.05$  in the human genome

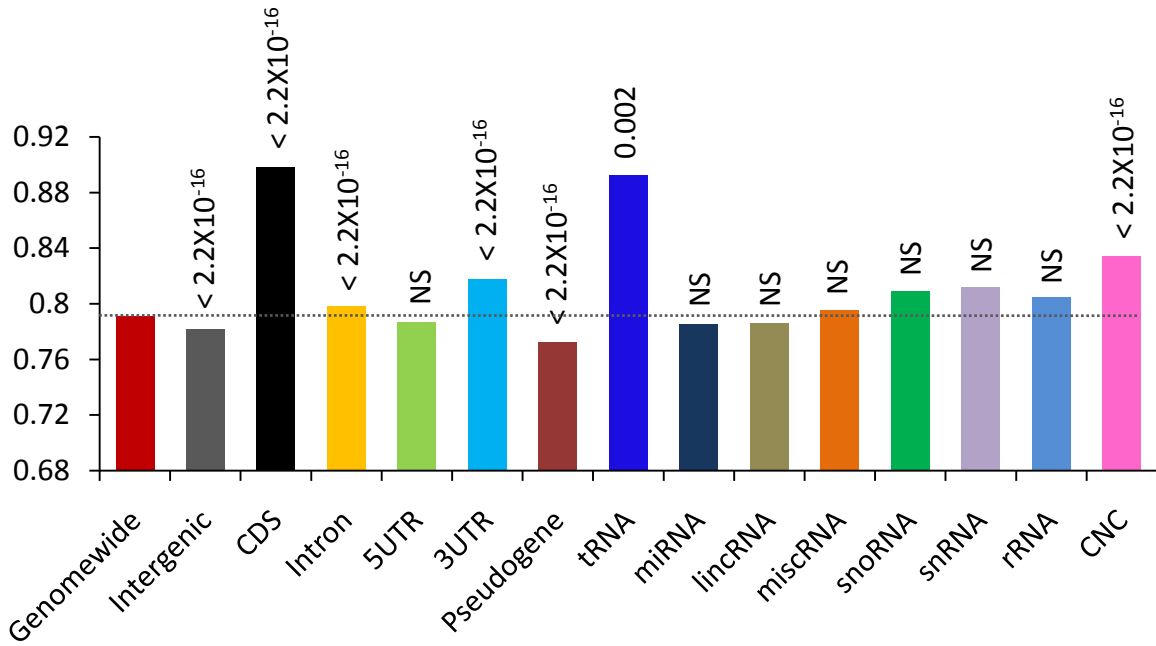

Supplement: S14 Fig — The number of low F ST variants is significantly high for CDS, Intron, 3′UTR, tRNA, and CNC but low for intergenic elements when compared to the genome-wide background. miscRNA, snoRNA, snRNA, and rRNA also showed a high number of such variants but the values obtained were not significant (χ2 test, P < 0.05). Also, 5′UTR showed less number of these variants but this difference in value from the genome-wide background was not significant. The dashed line indicates genome-wide threshold. (PDF) [file pone.0129023.s014.pdf]

**A**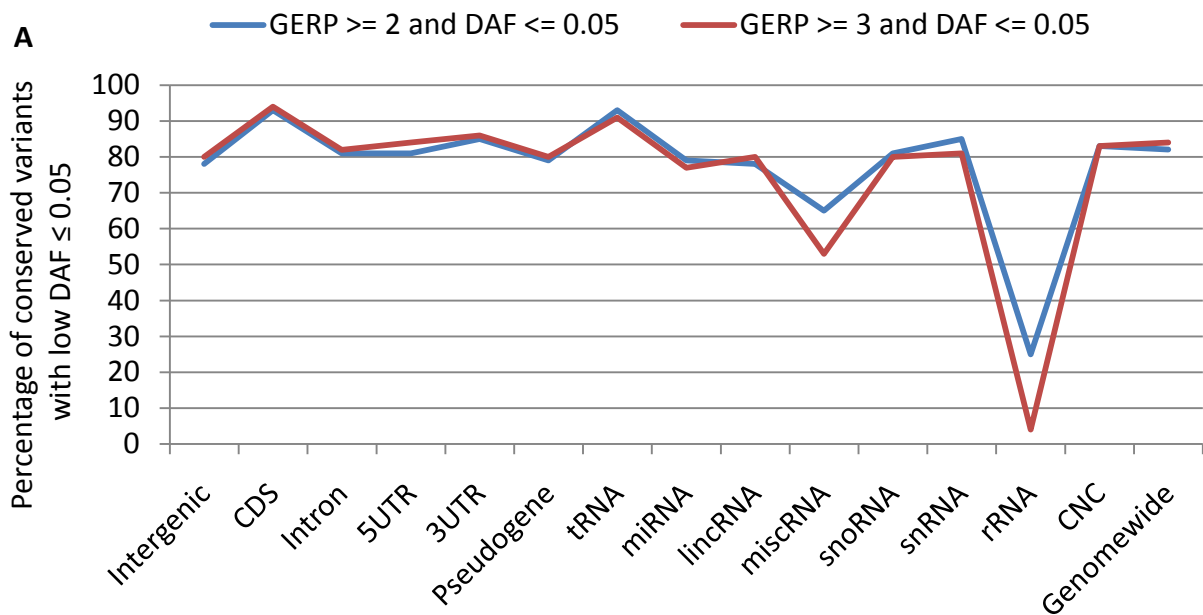**B**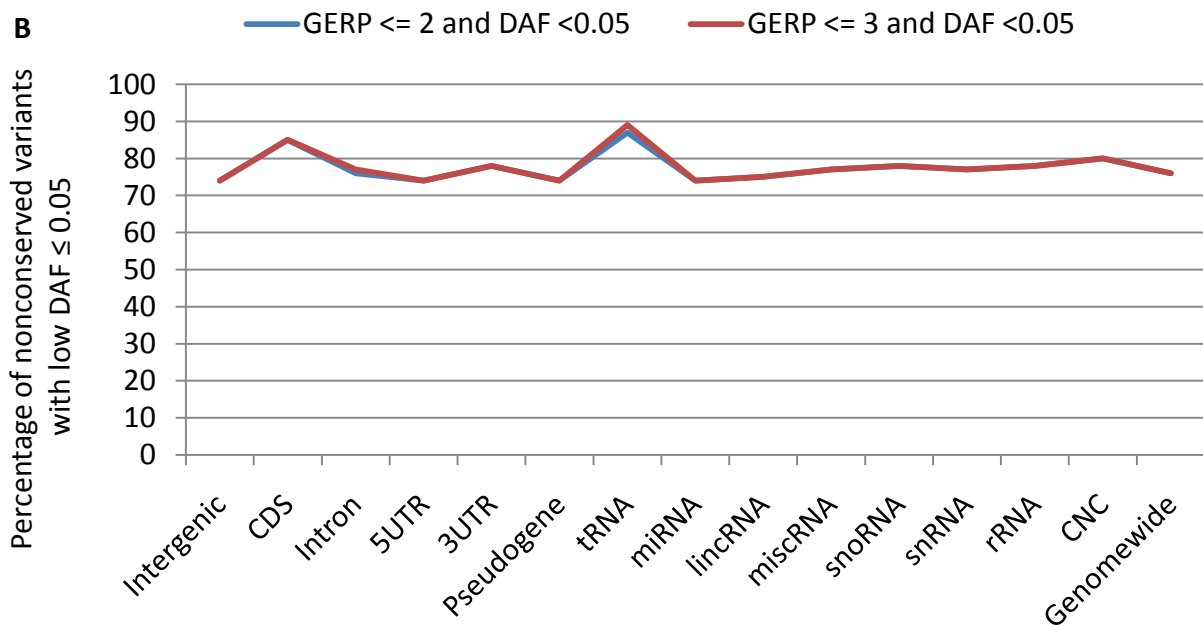

Supplement: S15 Fig — (PDF) [file pone.0129023.s015.pdf]

Fraction of variants having global  $F_{ST} > 0.4$  in the human genome

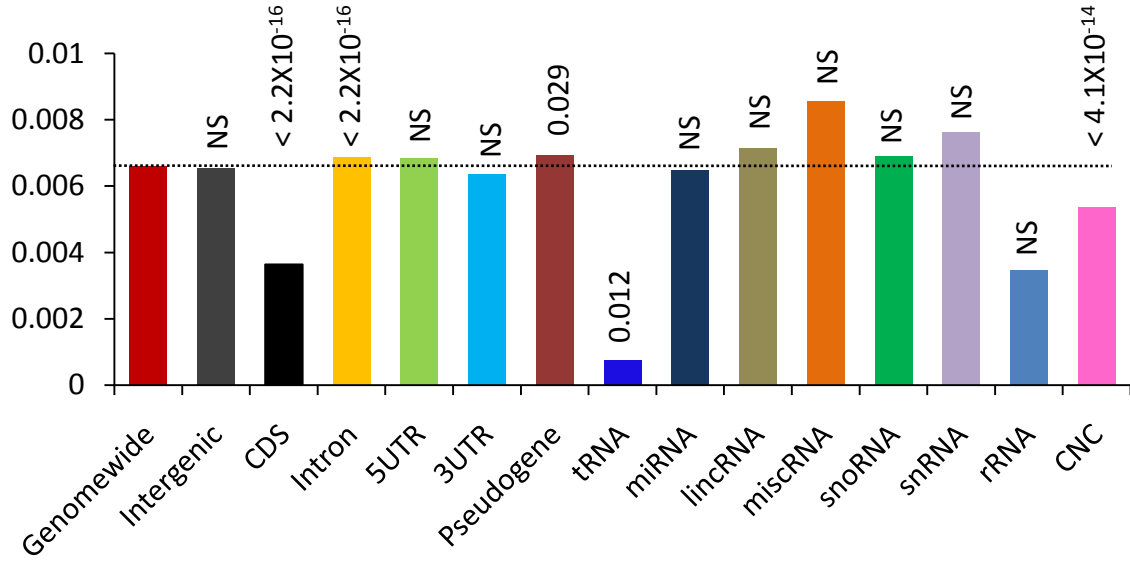

Supplement: S16 Fig — The number of high F ST variants is significantly higher for intron than that for the genome-wide background, whereas that for pseudogenes was marginally significant compared to the genome-wide background. However, CDS, tRNA, and CNC showed significant depletion for such variants. (PDF) [file pone.0129023.s016.pdf]

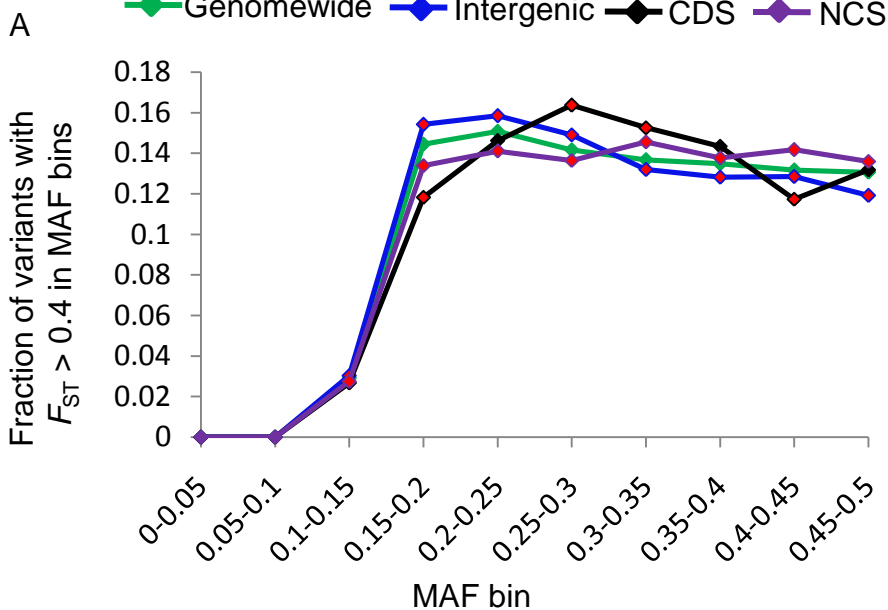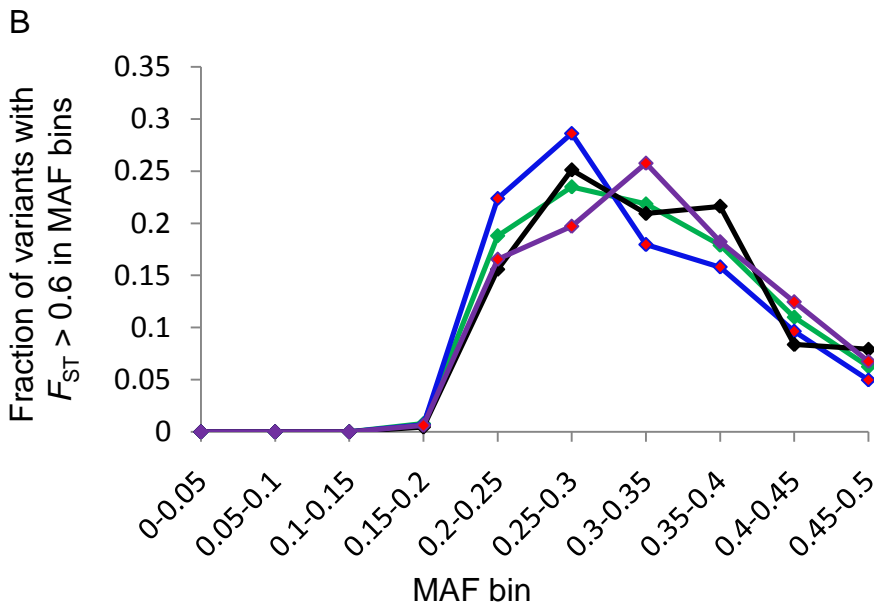

Supplement: S17 Fig — In this analysis, the number of high F ST variants was higher in NCS elements in different high MAF bins than in the genome-wide background when compared according to their respective bins (A) at > 0.4 (B) at > 0.6. (Red filled blocks represent significant differences: χ2 test, P< 0.05 used for all comparison, NS: not significant). (PDF) [file pone.0129023.s017.pdf]

**A**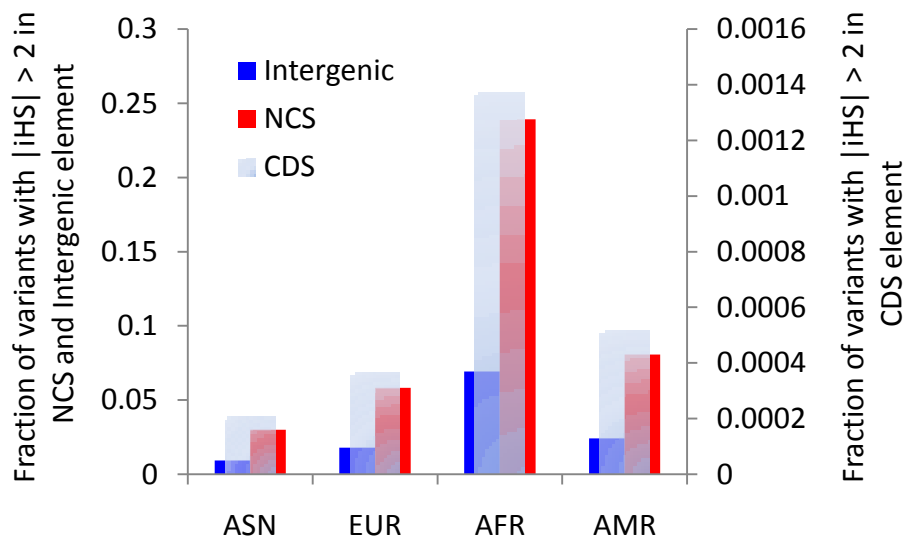**B**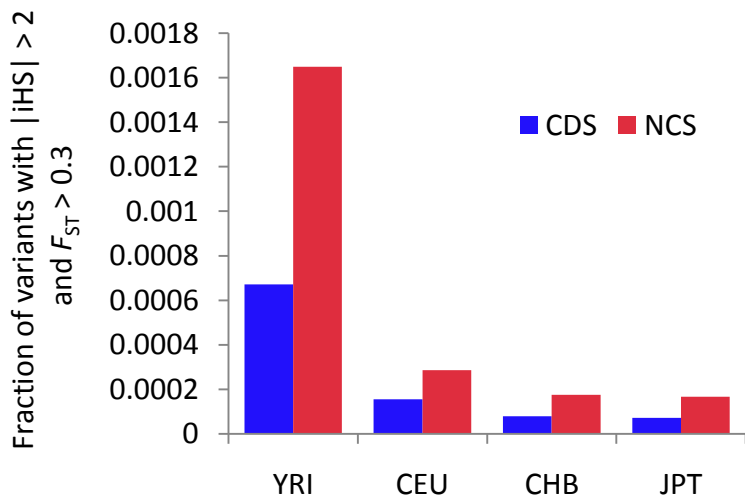

Supplement: S18 Fig — In our dataset, |iHS| score ≥ 2 variants showed significant enrichment in NCS sequences but depletion for CDS in the genome (χ2 test, P < 0.05) in all the populations (A). Also, with high |iHS| > 2 and high F ST > 0.3 variants, we detected a significantly enrichment for NCS sequences compared to CDS (χ2 test, P < 0.05) in all the populations (B). (PDF) [file pone.0129023.s018.pdf]

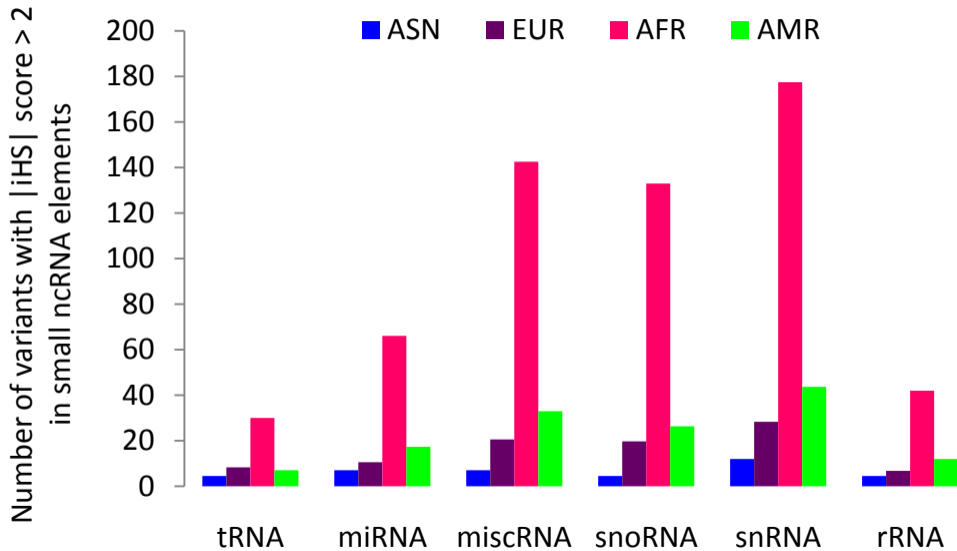

Supplement: S19 Fig — A significant number of ncRNAs variants were positive for the selection indicator, the |iHS| score in the human genome, and thus could have been a potential target for local adaptations. (PDF) [file pone.0129023.s019.pdf]

**A**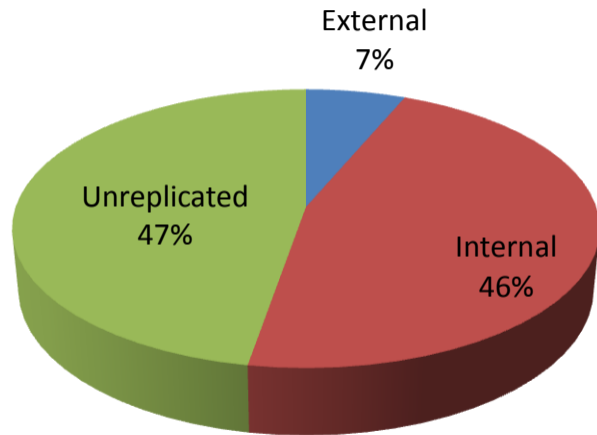**B**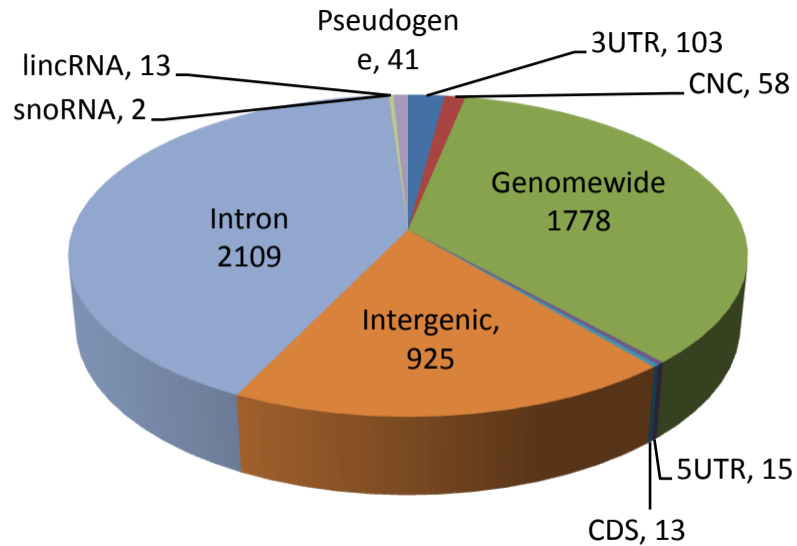

Supplement: S20 Fig — (A) Classification of validated associated variants from GWAS used in this analysis. (B) Distribution of GWAS variants in the various genomic elements. (PDF) [file pone.0129023.s020.pdf]

**A**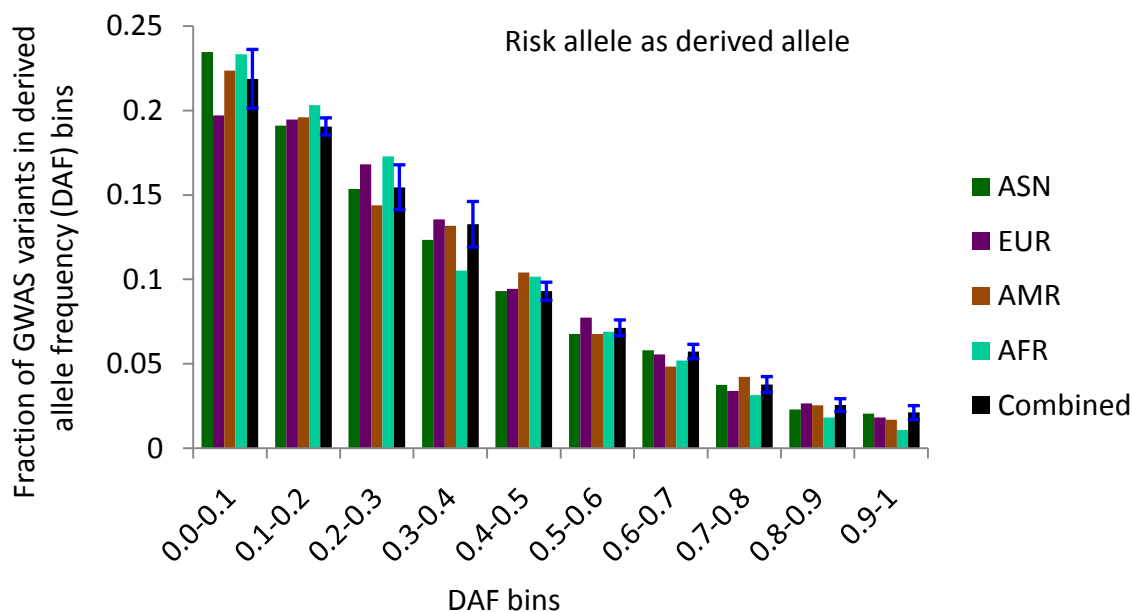**B**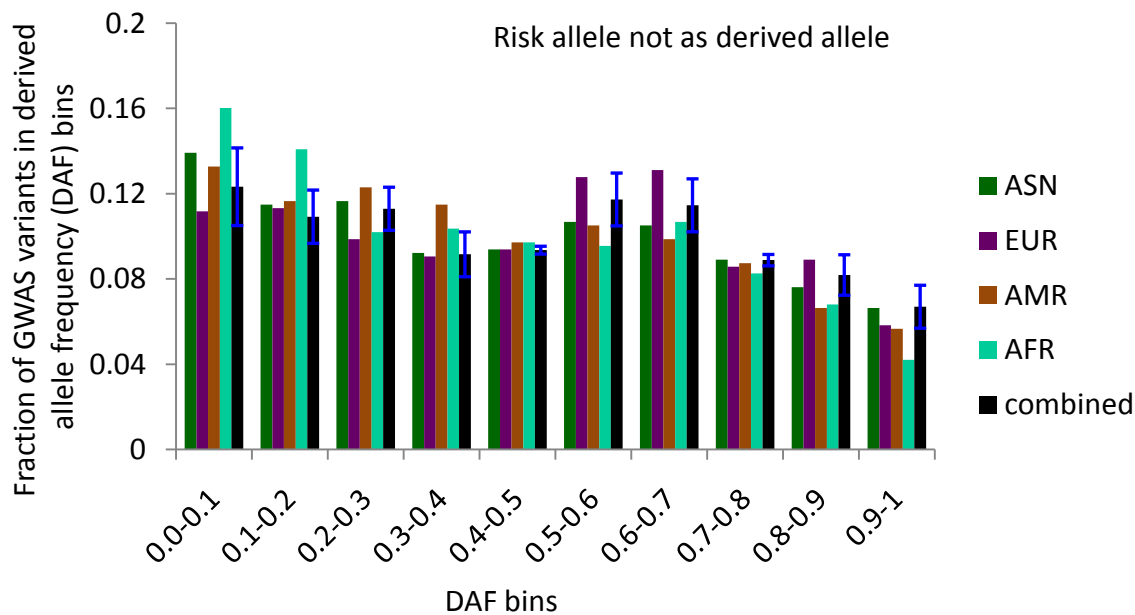

Supplement: S21 Fig — (A) Derived alleles which are risk alleles based on GWAS studies had frequency distribution restricted to lower DAF bins, (B) whereas derived alleles which are not risk alleles showed higher frequency in higher DAF bins. The combined data represents derived allele frequency across all the populations, and the error bar represents standard deviation. (PDF) [file pone.0129023.s021.pdf]

**A**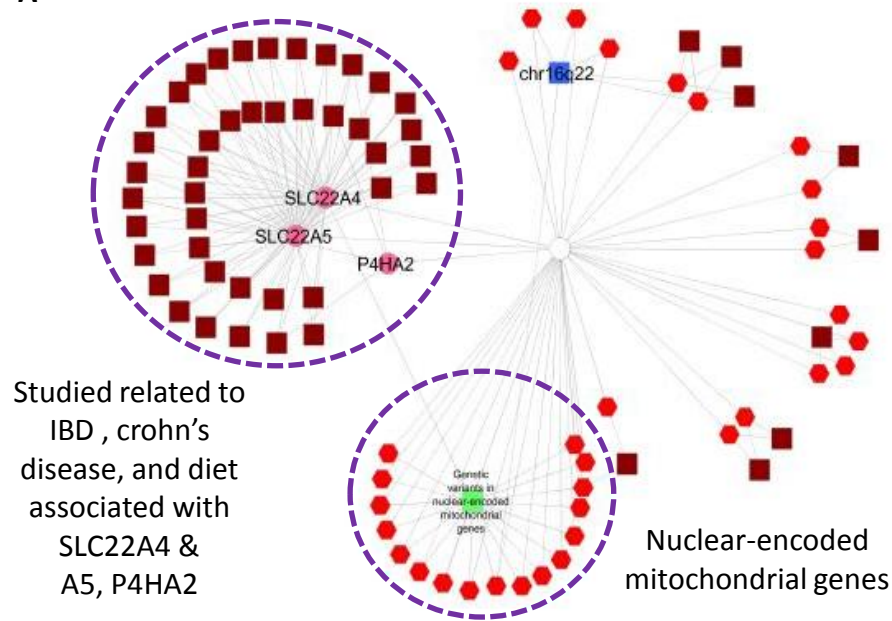**B**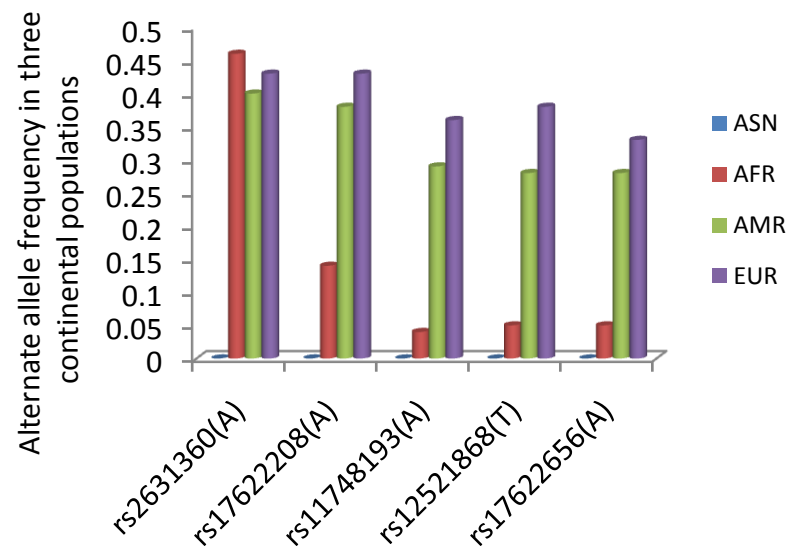**C**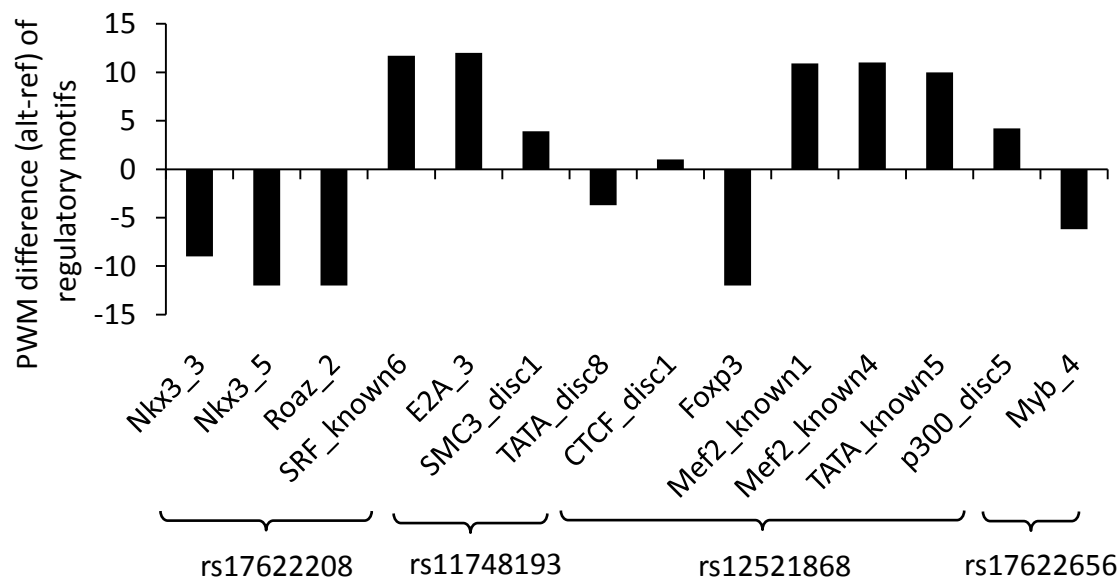

Supplement: S23 Fig — Gene set multi-feature enrichment (ToppCluster) in CEU of positively selected eQTL variants and their associated genes identified from eQTL browse and network drowns using Cytoscape software (A), Frequency of alternate alleles in continental populations (B), Log of position weight matrix (PWM) score for regulatory motifs for variants obtained from HaploRegV2 (C). (PDF) [file pone.0129023.s023.pdf]

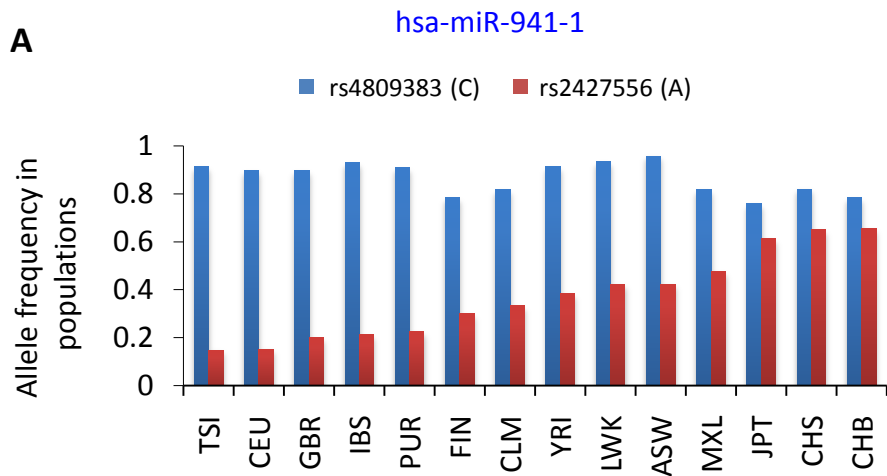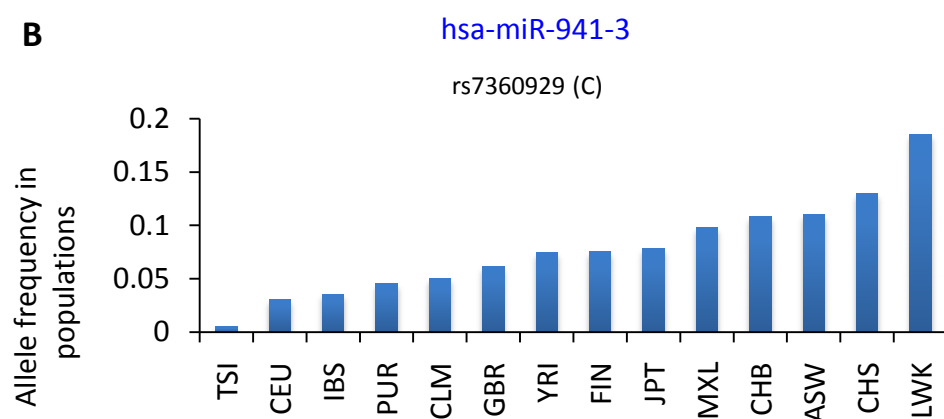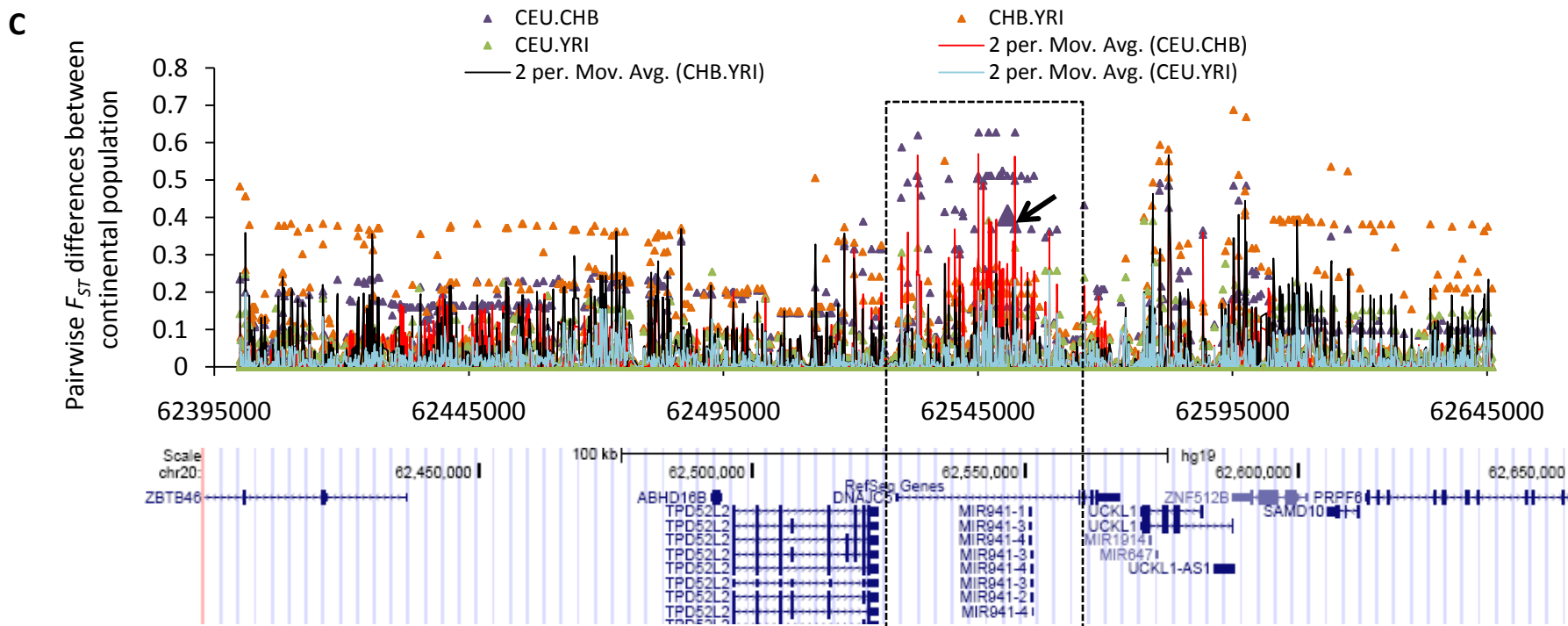

Supplement: S24 Fig — We identified rs2427556, located in the miR complementary region of miR-941-1 in this cluster (shown by dashed box in C), to be positive for the signature of selection based on F ST differences. (A) The moving average trend line shows the differences between CEU and CHB populations, and rs2427556 is shown by a black arrow. Two other variants were detected: rs4809383 is present in the stem region of mir-941-1 and rs7320929 is present in mir-941-3. (B) The allele frequency of rs2427556 was higher in Asian populations than in European populations, while rs4809383 has nearly the same frequency in worldwide populations. (C) The lower panel shows the UCSC genome browser view for the miR-941 cluster and iHS score. The X-axis shows the base position on chromosome 20 of the variants. (PDF) [file pone.0129023.s024.pdf]

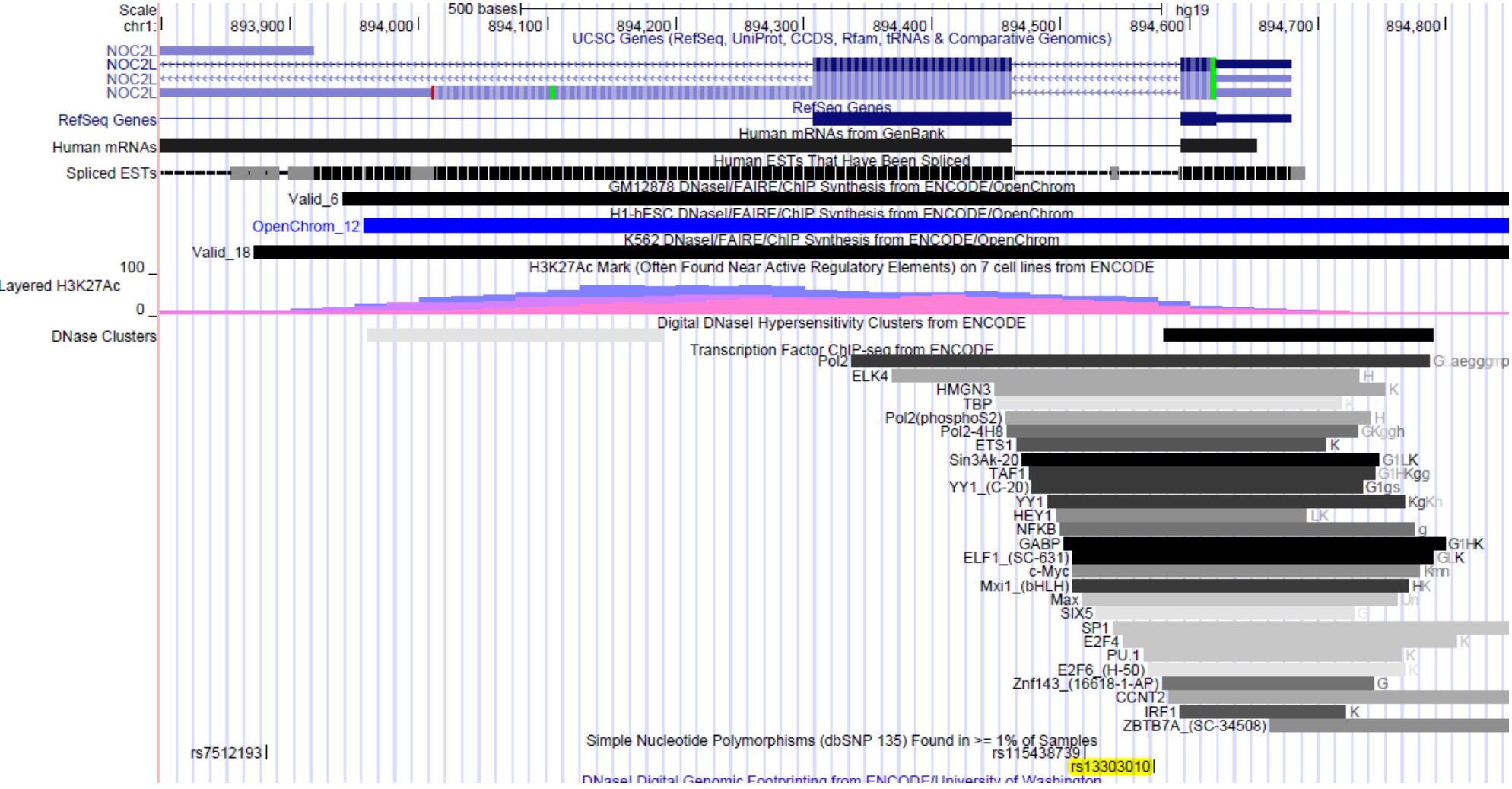

Supplement: S25 Fig — The SNP rs13303010, (marked in yellow) in an intron of NOC2L, was positive for a signature of selection that overlaps with several regulatory elements. Based on the top 1% high F ST > 0.3 cutoff, this variant showed differences between European and African populations. NOC2L is a novel HDAC-independent inhibitor of histone acetyltransferase (INHAT). Sequence variation in these TFs due to this SNP and gene expression by these TFs in the genome might perturb diverse biological functions in the regulatory network in a cell type-specific manner. (PDF) [file pone.0129023.s025.pdf]
