# Supplementary material for: Natural Selection and Functional Potentials of Human Noncoding Elements Revealed by Analysis of Next Generation Sequencing Data
Source: PLoS One. 2015 Jun 8;10(6):e0129023. doi: 10.1371/journal.pone.0129023 (PMC4460046; doi:10.1371/journal.pone.0129023)
Supplement: S11 Fig — Compared to the genome-wide background, intron, CDS, 5′UTR, 3′UTR, tRNA, and CNC had significantly enriched rare variants; pseudogene and intergenic element showed significant depletion; ncRNAs element showed population-specific significant enrichment for low DAF variants. (PDF) [file pone.0129023.s011.pdf]

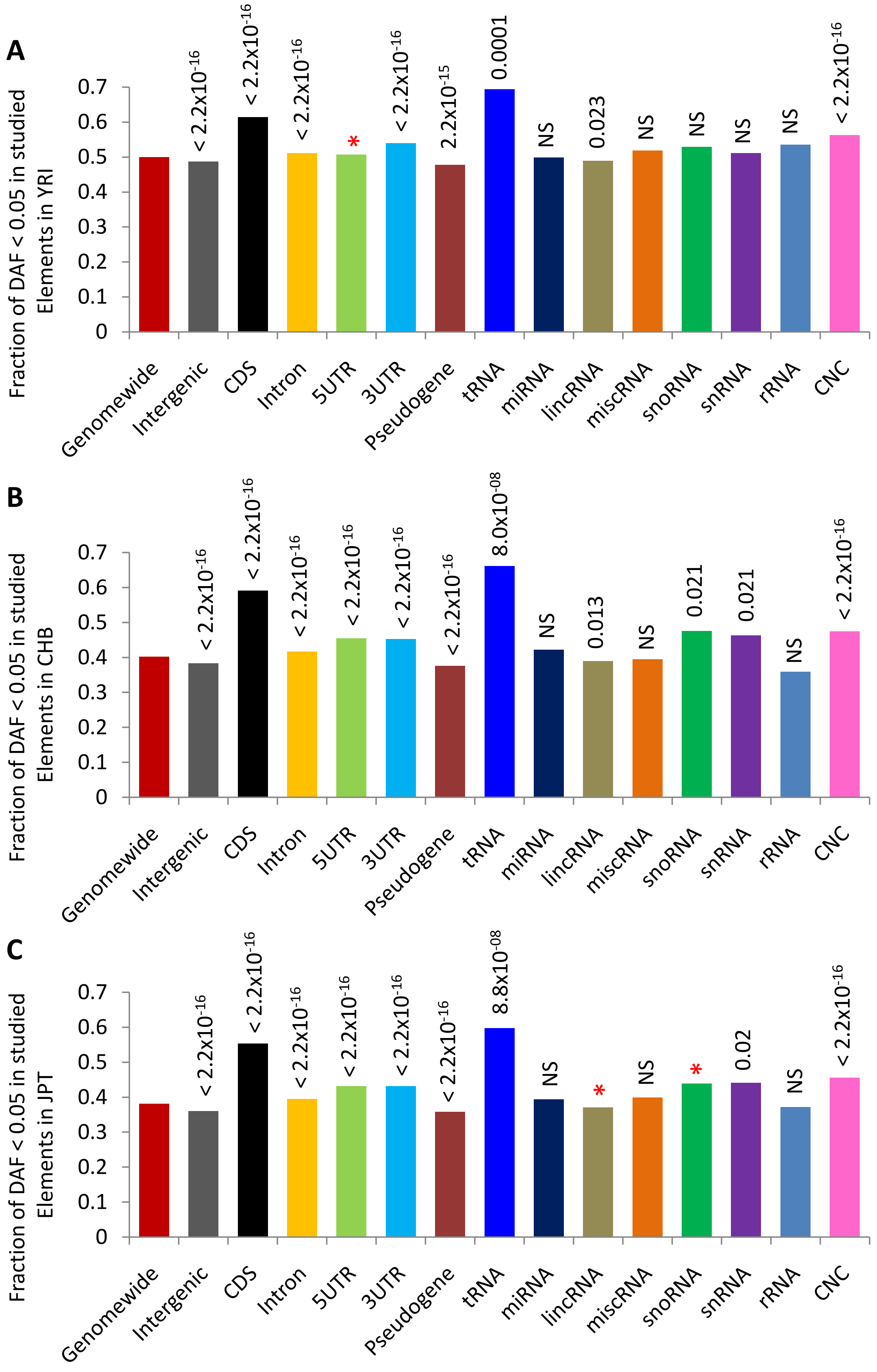
A
Fraction of DAF < 0.05 in studied Elements in YRI
0.7
0.6
0.5
0.4
0.3
0.2
0.1
0
< 2.2x10-16
< 2.2x10-16
< 2.2x10-16
*
< 2.2x10-16
2.2x10-15
0.0001
NS
0.023
NS
NS
NS
NS
< 2.2x10-16
Genomewide
Intergenic
CDS
Intron
5UTR
3UTR
Pseudogene
tRNA
miRNA
lincRNA
miscRNA
snoRNA
snRNA
rRNA
CNC
B
Fraction of DAF < 0.05 in studied Elements in CHB
< 2.2x10-16
< 2.2x10-16
< 2.2x10-16
< 2.2x10-16
< 2.2x10-16
< 2.2x10-16
8.0x10-08
NS
0.013
NS
0.021
0.021
NS
< 2.2x10-16
C
Fraction of DAF < 0.05 in studied Elements in JPT
< 2.2x10-16
< 2.2x10-16
< 2.2x10-16
< 2.2x10-16
< 2.2x10-16
< 2.2x10-16
8.8x10-08
NS
*
NS
*
0.02
NS
< 2.2x10-16
