# Supplementary material for: Natural Selection and Functional Potentials of Human Noncoding Elements Revealed by Analysis of Next Generation Sequencing Data
Source: PLoS One. 2015 Jun 8;10(6):e0129023. doi: 10.1371/journal.pone.0129023 (PMC4460046; doi:10.1371/journal.pone.0129023)
Supplement: S22 Fig — These variants are highly enriched for underlying regulatory markers for methylation, acetylation, DNase I, transcription factor binding sites, and protein bound motifs. These markers are represented accordingly in the three cell types. The differences in the patterns might be indicative of their roles in physiology and pathophysiology. (PDF) [file pone.0129023.s022.pdf]

NCS variants showed signature of positive selection in human popualtions enriched for regulatory marks and associated with phenotypes

SNP info		Disease	GM12878				K562				hESC			
elements	dbSNP	Traits	H3K4Me1	H3K4Me3	H3K27Ac	DNaseI	H3K4Me1	H3K4Me3	H3K27Ac	DNaseI	H3K4Me1	H3K4Me3	H3K27Ac	DNaseI
**Intronic**	rs2899472	AB1-42												
**non-Coding**	rs9331888	Alzheimer's disease												
**Intronic**	rs157580	Alzheimer's disease, cholesterol												
**Intronic**	rs13376333	Atrial fibrillation												
**Intronic**	rs7193343	Atrial fibrillation												
**3UTR**	rs3729931	Cardiac hypertrophy												
**Intronic**	rs17483466	Chronic lymphocytic leukemia												
**Intronic**	rs17293632	Crohn's disease												
**Intronic**	rs2188962	Crohn's disease												
**Intronic**	rs1378942	Diastolic blood pressure												
**Intronic**	rs471364	HDL cholesterol												
**Intronic**	rs7183263	Height												
**Intronic**	rs7507204	Height												
**Intronic**	rs1635852	Height												
**3UTR**	rs7097	Large B-cell lymphoma												
**Intronic**	rs36600	Lung cancer												
**5UTR**	rs2814778	Neutrophil count												
**Intronic**	rs2278729	Osteoporosis												
**Intronic**	rs2074518	QT interval												
**Intronic**	rs6741819	Response to antipsychotic treatment												
**Intronic**	rs174583	Response to statin therapy												
**Intronic**	rs174583	Response to statin therapy												
**Intronic**	rs589691	Serum urate												
**3UTR**	rs4763879	Type 1 diabetes												
