# Supplementary material for: Natural Selection and Functional Potentials of Human Noncoding Elements Revealed by Analysis of Next Generation Sequencing Data
Source: PLoS One. 2015 Jun 8;10(6):e0129023. doi: 10.1371/journal.pone.0129023 (PMC4460046; doi:10.1371/journal.pone.0129023)
Supplement: S5 Fig — For PCA analysis, resampled data of 870 markers were used from each element because rRNA has only 870 markers. The blue color represents European, red for Asian, black for African, and green for American populations. Data on American populations showed more closeness to European populations. (PDF) [file pone.0129023.s005.pdf]

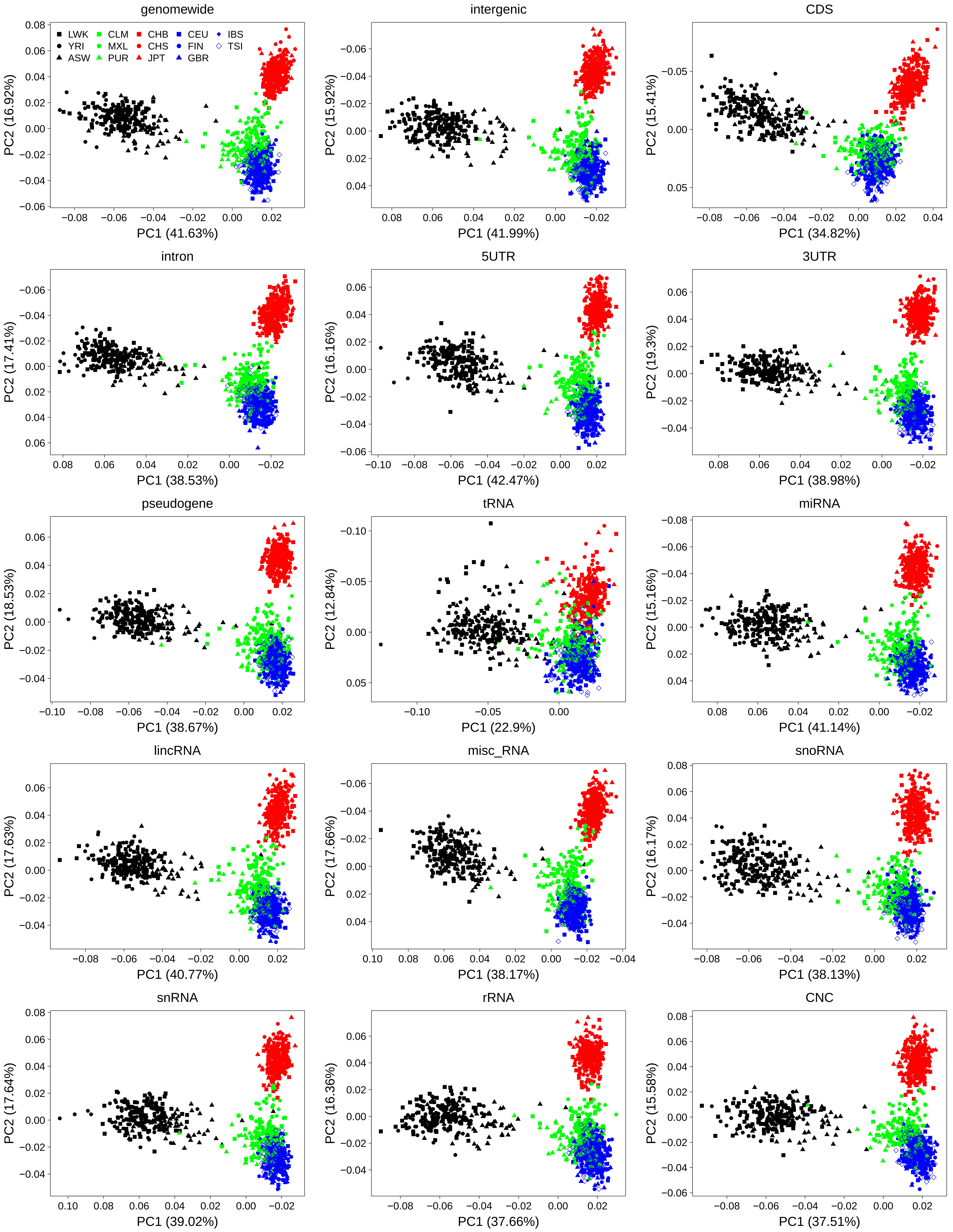

genomewide
LWK
YRI
ASW
CLM
MXL
PUR
CHB
CHS
JPT
CEU
FIN
GBR
IBS
TSI
PC1 (41.63%)
PC2 (16.92%)
intergenic
PC1 (41.99%)
PC2 (15.92%)
CDS
PC1 (34.82%)
PC2 (15.41%)
intron
PC1 (38.53%)
PC2 (17.41%)
5UTR
PC1 (42.47%)
PC2 (16.16%)
3UTR
PC1 (38.98%)
PC2 (19.3%)
pseudogene
PC1 (38.67%)
PC2 (18.53%)
tRNA
PC1 (22.9%)
PC2 (12.84%)
miRNA
PC1 (41.14%)
PC2 (15.16%)
lincRNA
PC1 (40.77%)
PC2 (17.63%)
misc_RNA
PC1 (38.17%)
PC2 (17.66%)
snoRNA
PC1 (38.13%)
PC2 (16.17%)
snRNA
PC1 (39.02%)
PC2 (17.64%)
rRNA
PC1 (37.66%)
PC2 (16.36%)
CNC
PC1 (37.51%)
PC2 (15.58%)
